# Supplementary material for: The functional brain favours segregated modular connectivity at old age unless affected by neurodegeneration
Source: Commun Biol. 2021 Aug 16;4:973. doi: 10.1038/s42003-021-02497-0 (PMC8367990; doi:10.1038/s42003-021-02497-0)
Supplement: Supplementary file 2 — Supplementary Information [file 42003_2021_2497_MOESM2_ESM.pdf]

Supplementary material for

The functional brain favours segregated modular connectivity at old age unless affected by neurodegeneration

Authors:

Xue Chen[1, 2, a, b], Joe Necus[2, 3, a, b], Luis R Peraza[4, 5, b], Ramtin Mehraram[2, 4, 6, 7, b], Yanjiang Wang[1], John T O'Brien[8], Andrew Blamire[4], Marcus Kaiser [2, 3, 4], John-Paul Taylor[4]

Affiliations

[1] *College of Control Science and Engineering, China University of Petroleum (East China), Qingdao, 266580, China.*

[2] *Interdisciplinary Computing and Complex BioSystems (ICOS) research group, School of Computing, Newcastle University, Newcastle upon Tyne, NE4 5TG, United Kingdom.*

[3] *University of Nottingham, NIHR Nottingham Biomedical Research Centre, School of Medicine, Nottingham, UK.*

[4] *Translational and Clinical Research Institute, Newcastle University, Campus for Ageing and Vitality, Newcastle upon Tyne, NE4 5PL, United Kingdom*.

[5] *IXICO Plc, London, UK*

[6] *Experimental Oto-rhino-laryngology (ExpORL) Research Group, Department of Neurosciences, KU Leuven, Leuven, Belgium.*

[7] *NIHR Newcastle Biomedical Research Centre, Campus for Ageing and Vitality, Newcastle upon Tyne, UK*

[8] *Department of Psychiatry, University of Cambridge School of Medicine, Cambridge, CB2 0SP, United Kingdom.*

Emails

Xue Chen: wmtmdlove@163.com

Joe Necus, Email: Joseph.necus@nottingham.ac.uk

Luis R. Peraza: luis.peraza@gmail.com

Ramtin Mehraram: ramtin.mehraram@kuleuven.be

Yanjiang Wang: yjwang@upc.edu.cn

John T O'Brien: john.obrien@medschl.cam.ac.uk

Andrew Blamire: andrew.blamire@newcastle.ac.uk

Marcus Kaiser: Marcus.Kaiser@nottingham.ac.uk

John-Paul Taylor: john-paul.taylor@newcastle.ac.uk

Footnote:

[b] Equally Contributing authors

[a] Corresponding authors:

Xue Chen. College of Control Science and Engineering, China University of Petroleum (East China), No.66 Changjiang West Road, Huangdao district, Qingdao, China, 266580. Email: wmtmdlove@163.com;

Joe Necus. NIHR Nottingham Biomedical Research Centre, School of Medicine, University of Nottingham, Nottingham, UK. Email: Joseph.necus@nottingham.ac.uk;

This PDF file includes

- Supplementary Table 1 to Supplementary Table 2
- Supplementary Fig 1 to Supplementary Fig 22
- Supplementary Discussion: Validation analysis
- Supplementary Discussion: Motion analysis
- Supplementary Discussion: Standard parcellation analysis
- Supplementary Discussion: Limitations and considerations
- Supplementary References

Supplementary Table 1. Study demographics.

	NKI		TFC		NCL			
	YA	OA	YA	OA	ADD	DLB	PDD	OA
N=787	N=151	N=146	N=257	N=102	N=42	N=38	N=17	N=34
Age	27.1(5.4)	68.7(5.0)	22.9(4.35)	58.7(9.0)	77.2(8.6)	77.6(6.4)	71.8(5.0)	76.4(7.0)
Sex	74M,77F	48M,98F	102M,145F	44M,58F	30M,12F	25M,13F	17M,0F	24M,10F
MMSE	--	--	--	--	21.2(3.7)	21.6(4.6)	24.3(2.9)	28.9(1.1)
CAMCOG	--	--	--	--	68.8(12.9)	72.31(14.5)	79.5(7.36)	96.56(3.4)
UPDRS III	--	--	--	--	3.7(3.6)	18.1(10.31)	25.7(7.16)	1.97(2.8)

All values are shown as mean (standard deviation)
N, number of participants; M, male; F, Female
MMSE, Mini-mental state examination
CAMCOG, Cambridge cognition examination
UPDRS III, Unified Parkinson's disease rating scale III
YA, young adults
OA, older adults
ADD, Alzheimer's disease dementia
DLB, dementia with Lewy bodies
PDD, Parkinson's disease dementia

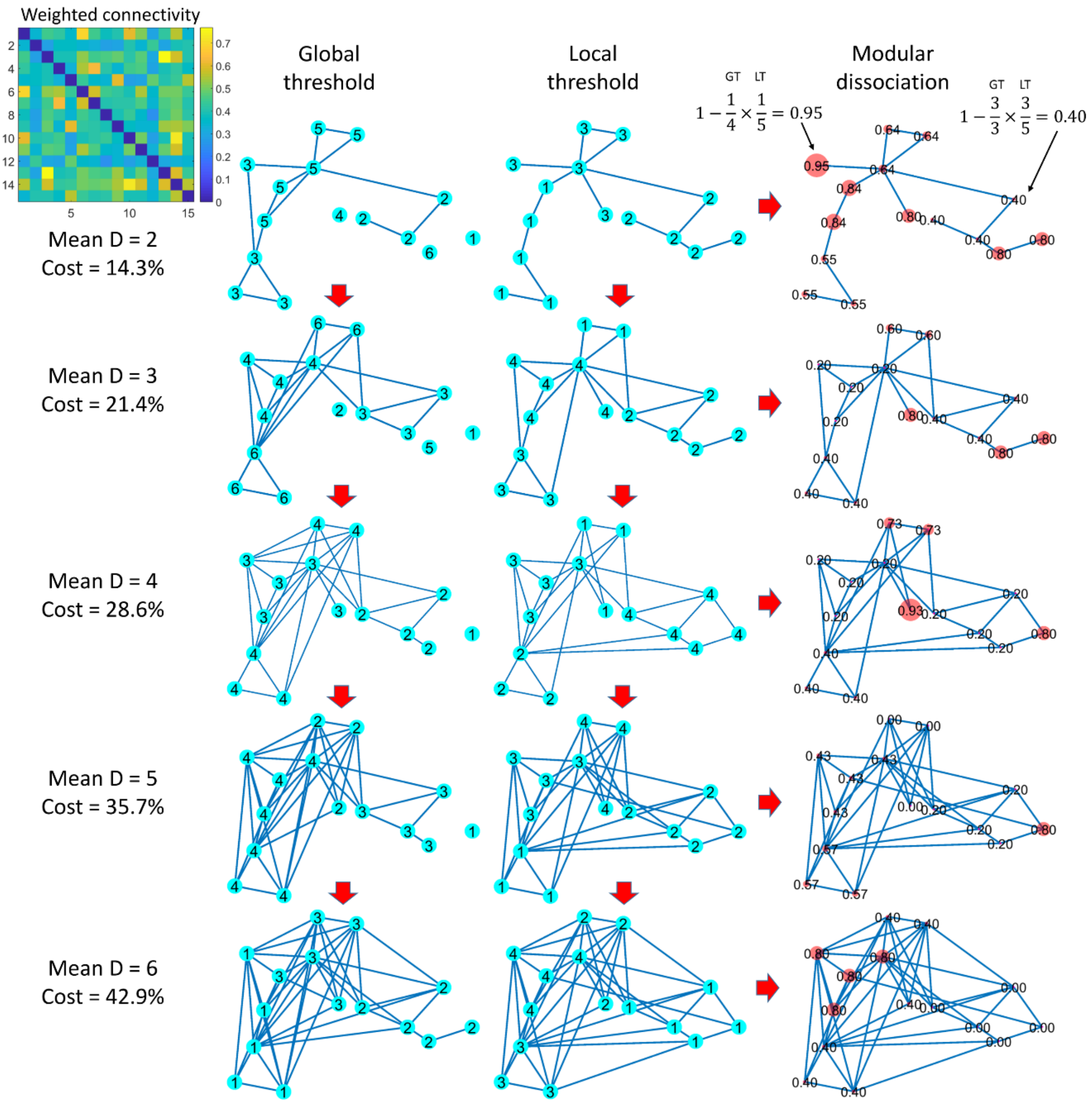

Supplementary Fig 1. Global threshold (GT) and local threshold (LT) network construction methods. The figure shows examples for both network construction methods in a proportionally thresholded network at five densities (cost) defined by the mean node degree D. Modularity was estimated with Louvain's algorithm (100 iterations for this example only) and estimated communities are numbered within each node and network. Results for modular dissociation (MD) are shown for the variability between globally and locally thresholded matrices. In the top row, two MD computation examples are given; first, modules 3 (GT with 4 nodes) and 3 (LT with 5 nodes) have one node in common which leads to an MD = 0.95. Second, modules 2 (GT with 3 nodes) and 2 (LT with 5 nodes) have three nodes in common which lead to an MD= 0.40.

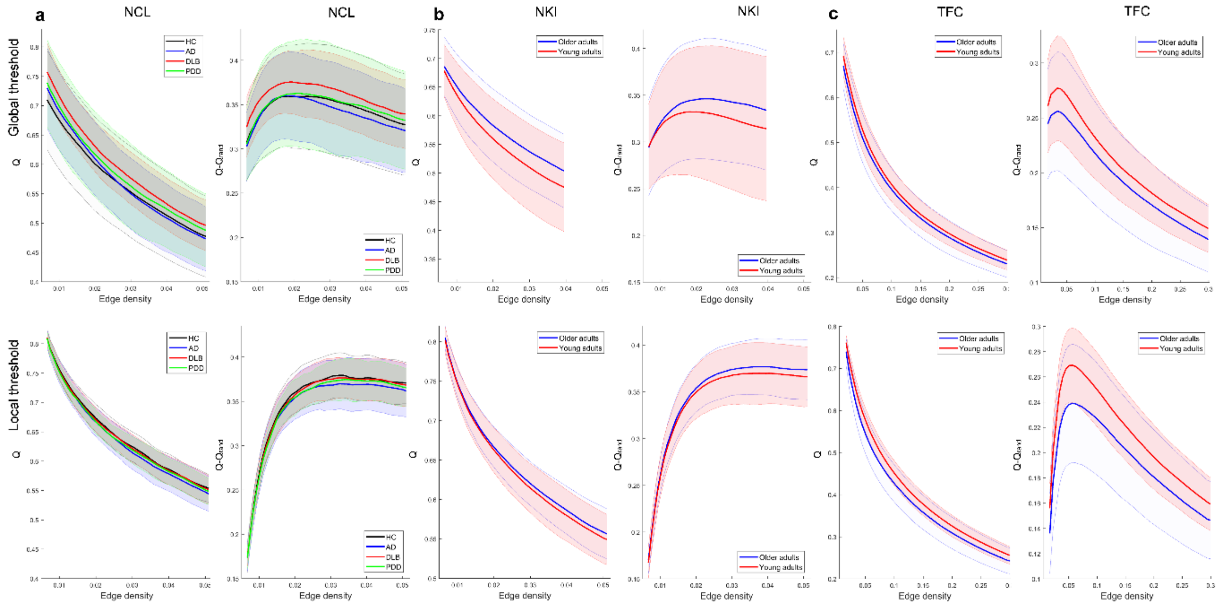

Supplementary Fig 2: Modularity across a range of edge densities and optimal edge density discovery. For the three cohorts in this study, the optimal local threshold was estimated based on the edge density that maximised modularity statistic Q when compared with the modularity statistic of an equivalent random network (normalised modularity $Q\text{-}Q_{rand}$). Q_{rand} was estimated as the average Q from 40 equivalent random networks. **a** Newcastle University (NCL) cohort, modularity and normalised modularity estimated with global (top) and local (bottom) threshold network construction methods; optimal densities reached at 1.9% (global) and 3.24% (local). **b** Same as (**a**) for the NKI cohort; optimal densities were reached at ~2.04% (global) and 3.8% (local). **c** Same as (**a)** for the 1000 functional connectome (TFC) cohort; optimal edge densities were reached at 3.39% (global) and 5.11% (local). Only the NKI cohort with global construction did not reach optimal density at the same value for both groups; for older adults, this was reached at 2.26% and for young adults at 1.81%.

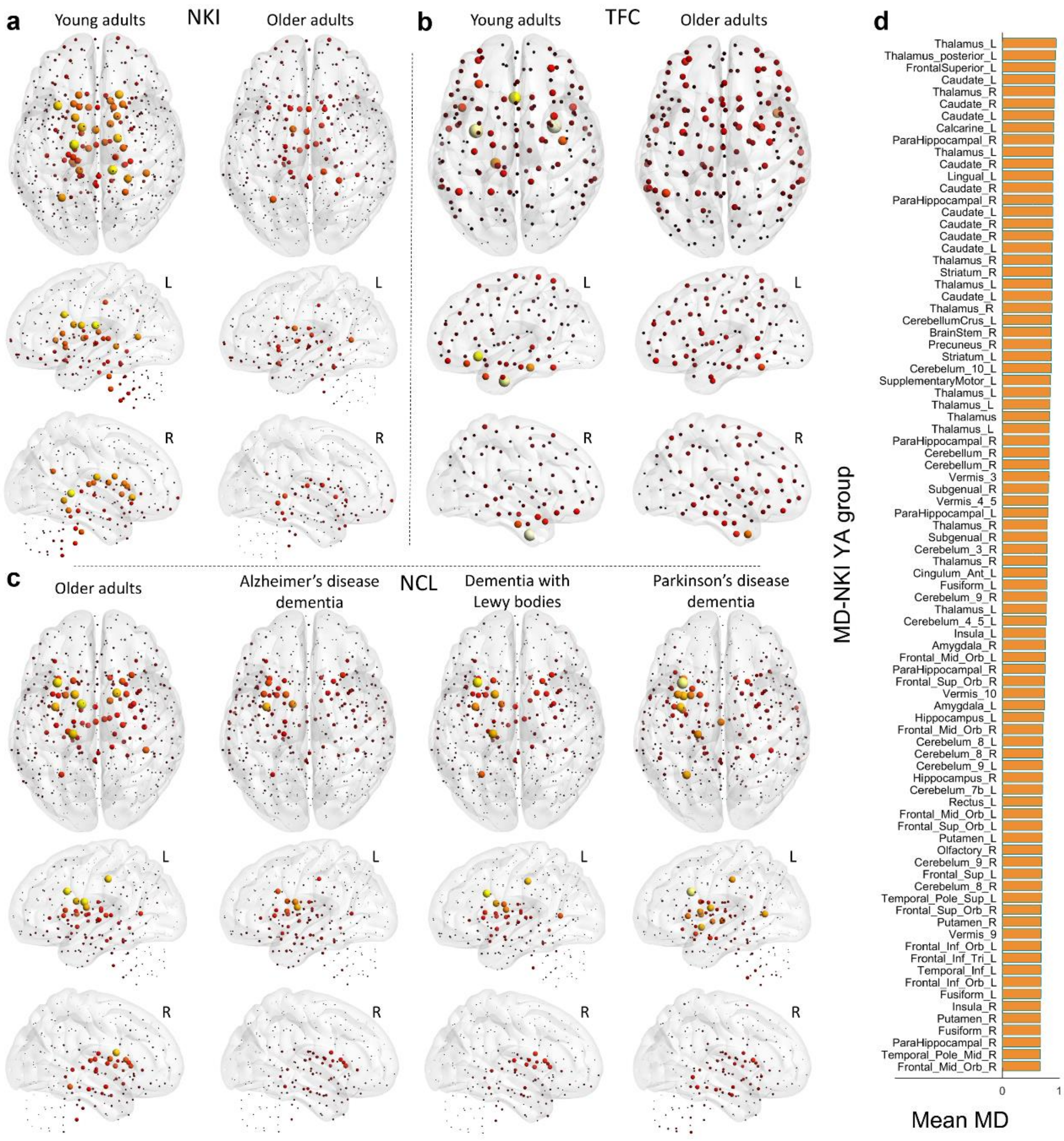

Supplementary Fig 3. Mean modular dissociation (MD) for all study groups. **a** Nathan Kline Institute (NKI) cohort MD values for the young adult (YA) and older adult (OA) groups. **b** Same as (**a**) for the 1000 functional connectome (TFC) cohort. **c** Same as (**a**) for the Newcastle (NCL) cohort. **d** Strongest 20% of MD values within the NKI-YA group. Left hemisphere (L), right hemisphere (R). Results presented are from the 451-ROI atlas at optimal edge density; for results using other atlases and densities see Supplementary Fig 6.

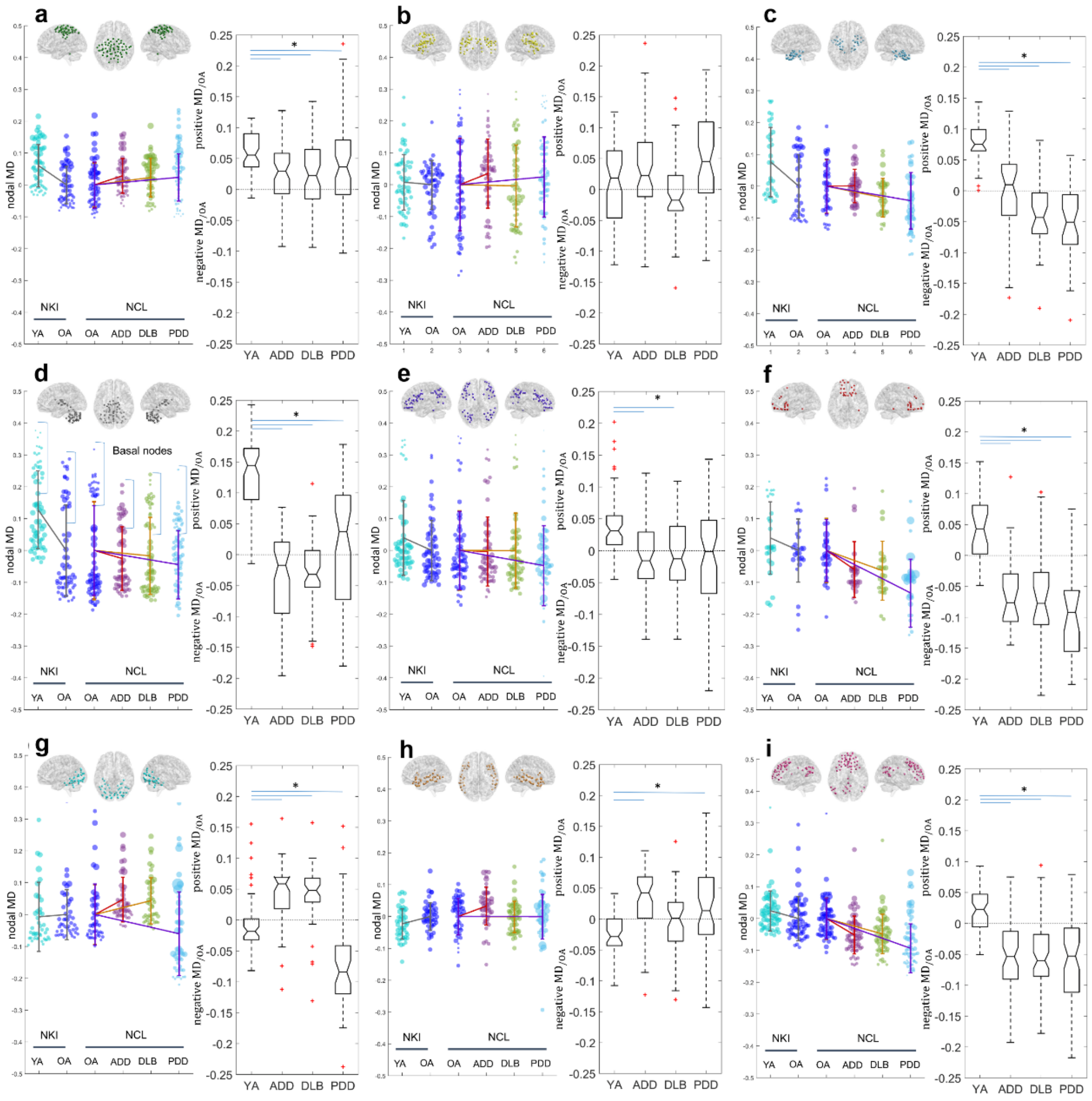

Supplementary Fig 4. Modular Dissociation (MD) values from the Nathan Kline Institute (NKI) and Newcastle University (NCL) databases. MD values are shown as dotted violin plots; regressed vectors *[R1 R2]-E(R1)* where *E* stands for expected value (see materials and methods in the main manuscript). The size of each dot (which represents a node) is proportional to the group MD variance. **a** motor-sensory, **b** Cingulo-Insular, **c** Temporal, **d** Cerebello-Basal, **e** Lateral Frontal-Parietal, **f** Ventral-Frontal, **g** Occipital, **h** Insular-Occipital, **i** Cerebello-basal. **d** The nodes within the cerebello-basal module showed a bimodal distribution dictated by the higher MD in basal nodes and lower MD in cerebellar nodes. Box plots with $MD_{/OA}$ values are also shown for each module. The mean values and standard deviations are derived from the whole module across nodes within the module.

Supplementary Discussion

Validation analysis

The previous set of experiments were implemented using 451-ROI atlas at optimal edge density; the density at which the modularity index difference $Q - Q_{rand}$ was found to be maximum (Supplementary Fig 2). However, in order to observe if our analyses were also valid at other densities, we estimated MV and MD at 10% and 20% edge density. Additionally, three brain parcellations were implemented in addition to the 451-ROI one; these were 100, 200 and 247 ROIs. Results from this validation analysis are presented as supplementary material. Supplementary Fig 5 shows validation results on the NKI database for the mean group MV using three functional parcellation resolutions; 100, 200 and 247 ROIs, all of them at their respective optimal edge density. For both groups, OA and YA, the same patterns are previously seen in Fig 3 in the main text are reproduced regardless of the number of ROIs. Supplementary Fig 5g-l shows results for MV at 451-ROI resolution at 10% and 20% densities. Again, the pattern of higher MV within parietal, insular and inferior temporal cortices is reproduced in both groups, YA and OA. Validation of MV on the NCL cohort showed similar patterns (not published).

Validation results for MD in the NCL cohort using the different atlas parcellations and edge densities are shown in Supplementary Fig 6. Here, there was a high variability on MD amplitude values across the range of densities and parcellations, however, the pattern of higher MD at insular and basal regions and lower MD at motor-sensory, occipital cortices and cerebellum remained invariant. Additionally, high contrast between regions of high and low MD is seen for results at optimal edge density. Validation on the NKI cohort showed similar patterns (not published) and this analysis was not implemented on the TFC cohort because only connectivity matrices at a fixed parcellation were available for this cohort.

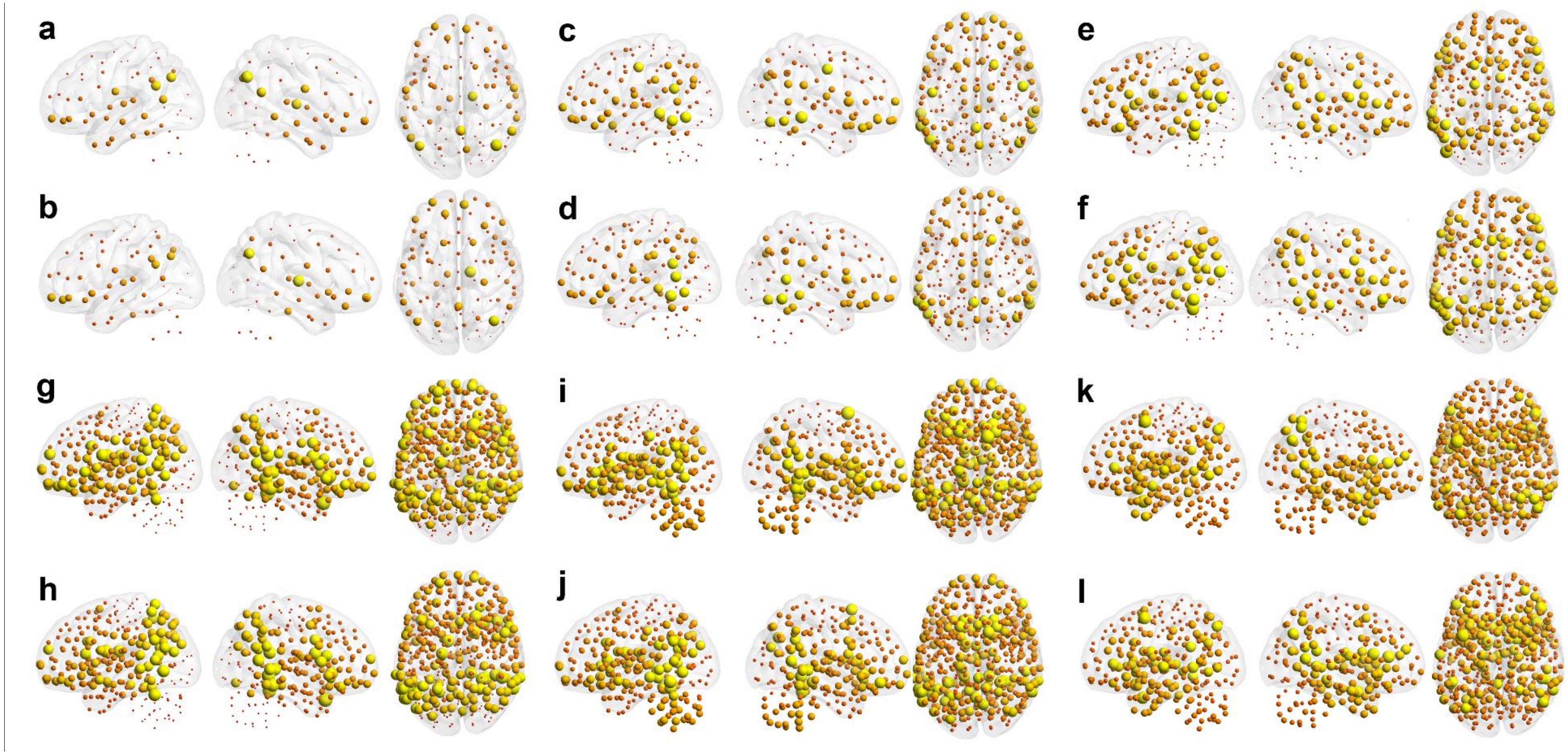

Supplementary Fig 5. Validation results for modular variability (MV) at different resolutions (number of regions) and edge densities for the NKI database. **a** Mean MV in older adults (OA) with 100 regions of interest (ROI) atlas at optimal edge density 10%. **b** Mean MV in young adults (YA) using a 100-ROI atlas at optimal density 10%. **c** Mean MV in OAs with 200-ROI atlas at optimal edge density 6.53%. **d** Mean MV in YAs with 200-ROI atlas at optimal density 6.53%. **e** Mean MV in OAs with 247-ROI atlas at optimal density 5.28%. **f** Mean MV in YAs with 247-ROI atlas at optimal density 5.28%. **g**, **i**, **k** Mean MV in OA with 451-ROI atlas at optimal edge density 3.8%, 10% and 20%. **h**, **j**, **l** Mean MV in YA with 451-ROI atlas at optimal density 3.8%, 10% and 20%.

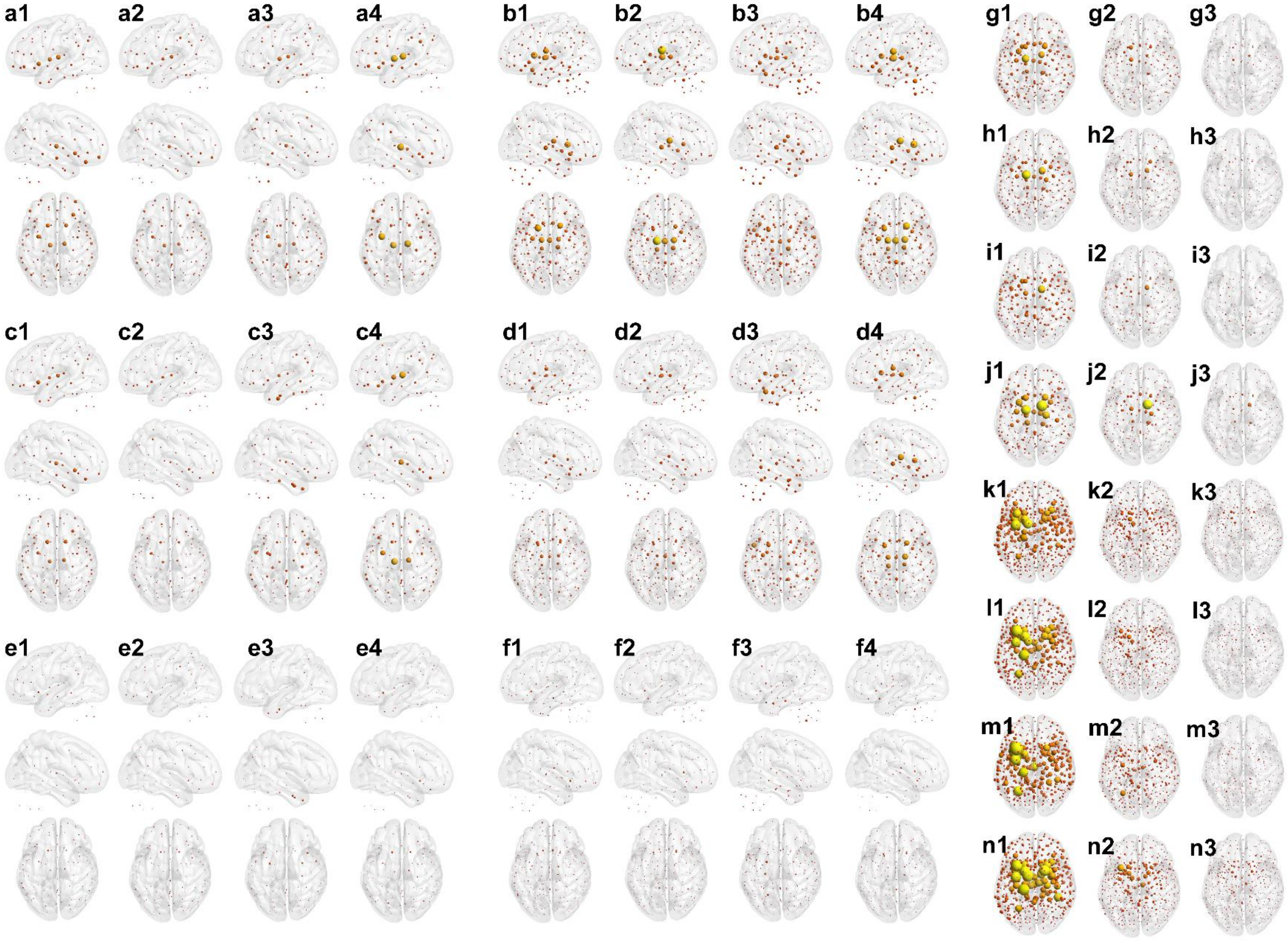

a1
a2
a3
a4
b1
b2
b3
b4
g1
g2
g3
h1
h2
h3
i1
i2
i3
c1
c2
c3
c4
d1
d2
d3
d4
j1
j2
j3
k1
k2
k3
e1
e2
e3
e4
f1
f2
f3
f4
l1
l2
l3
m1
m2
m3
n1
n2
n3

Supplementary Fig 6. Validation of modular dissociation (MD) results across different parcellation resolutions (atlases) and densities; optimal edge density, 10% and 20% of the strongest edges within the Newcastle University (NCL) database. **a1**-**a4**) 100-ROI atlas at optimal edge density of 6%, **a1**) older adults (NCL healthy controls), **a2** Alzheimer's disease dementia (ADD), **a3** dementia with Lewy bodies (DLB) and **a4** Parkinson's disease dementia (PDD). **c** and **e** same as (**a**) for 10% and 20% edge density respectively same 100-ROI atlas. **b1**-**b4** 200-ROI atlas at optimal edge density of 6%, **b1** older adults, **b2** ADD, **b3** DLB, **b4** PDD. **d** and **f** the same as (**b**) for densities 10% and 20%. **g1**-**g3** ADD MD values for the 247-ROI atlas at an optimal density of 5.28%, 10% and 20% in the same order. **h1**-**h3** same as **g1**-**g3** for the DLB group. **i1**-**i3** same as **g1**-**g3** for the PDD group. **j1**-**j3** same as **g1**-**g3** for the older adult (NCL healthy control) group. **k1**-**k3** ADD MD values for the 451-ROI atlas at an optimal density of 3.24%, 10% and 20% in the same order. **l1**-**l3** Same as **k1**-**k3** for the DLB group. **m1**-**m3** same as **k1**-**k3** for the PDD group. **n1**-**n3** Same as **k1**-**k3** for the older adult (NCL healthy control) group.

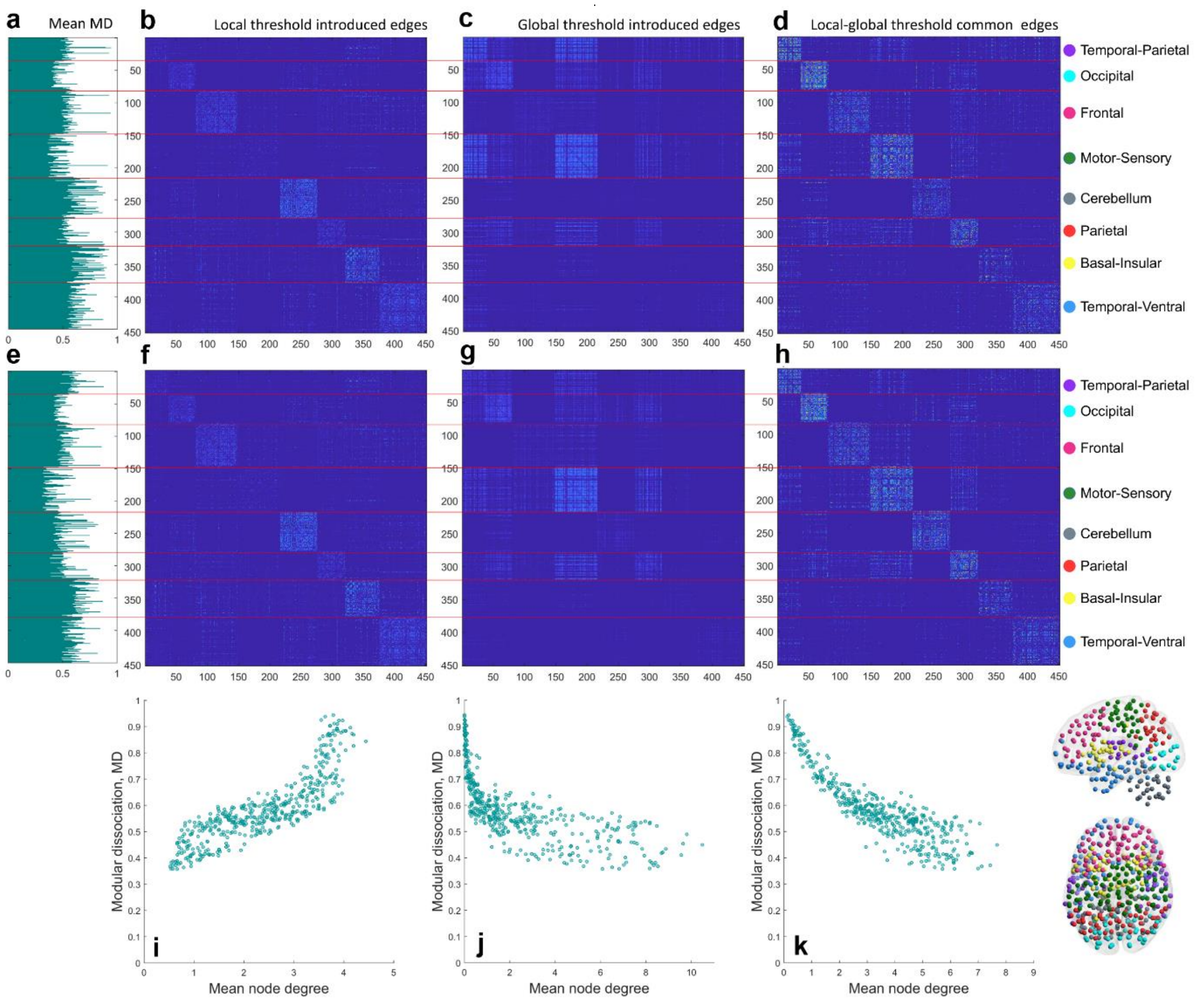

Supplementary Fig 7. Edge influence from both network construction methods, local and global threshold methods on the modular dissociation (MD) index. **a** Mean nodal MD index for the NKI young adult (YA) group (451 regions of interest). Mean MD values are presented in a vertical bar plot (451 bars accounting for all nodes) and ordered by modules. **b** Edges introduced by local threshold at optimal edge density. **c** Edges introduced by global threshold at optimal edge density (module definition follows the local threshold method). **d** Edges introduced by both local and global network construction methods. **e**-to-**h** same as above for the older adult (OA) group within the NKI cohort. **i**, **j**, **k** Influence (Spearman correlation) of the mean node degree for edges introduced by both network construction methods on the nodal MD values. **i** By local threshold r=0.884 p-value=1.30e-150. **j** By global threshold: r=-0.8416, p-value=3.323e-122, **k** By common edges r=-0.884, p-value=2.51e-150.

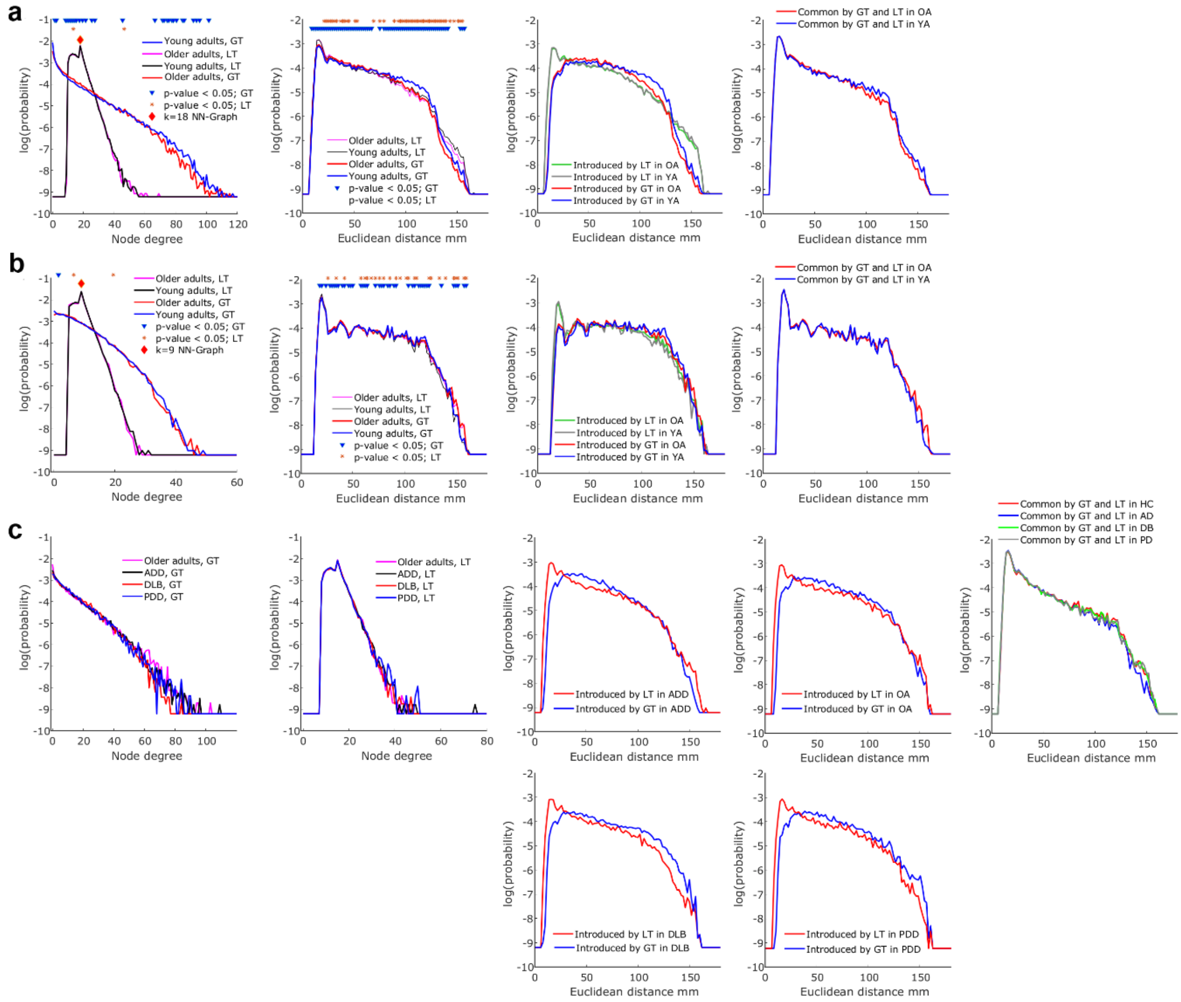

Supplementary Fig 8. Node degree and edge Euclidean distance distributions. **a** NKI cohort. **b** TFC cohort. **c** NCL cohort. First column: No degree by global and local threshold construction methods. Second column: Euclidean edge distribution by global and local threshold at optimal edge density. Third column: Introduced edges by local and global threshold only. Note that local threshold tended to introduce weaker close-range connections although long-range connections were also present and with higher probability than in global threshold network construction. Last column: Edges that were introduced by both local and global threshold.

Motion analysis

Participant motion within the MRI scanner may drive spurious results when comparing young vs older adults; it is known that older adults tend to move more than young adults during acquisition of neuroimages. We assessed motion by estimating Framewise displacement, *FD*[1] which integrates the six motion parameters estimated during the motion correction step by FSL-FLIRT. Framewise displacement is defined as $FD_i = |\Delta x_i| + |\Delta y_i| + |\Delta z_i| + |\Delta a_i| + |\Delta b_i| + |\Delta c_{ix}|$, where $\Delta x_i = x_{i-1} - x_i$ and similarly for the other rigid body parameters $[y_i, z_i, a_i, b_i, c_i]$. Rotational displacement was calculated on a surface of a 65-mm radius sphere.

Supplementary Fig 9 shows mean FD values (mean across time *i*) for all participants and groups. The 1000 functional connectome cohort (TFC) was not assessed because this cohort was pre-processed in a previous study[2], and motion parameters were not available. Only the Newcastle (NCL) and the NKI cohorts were studied.

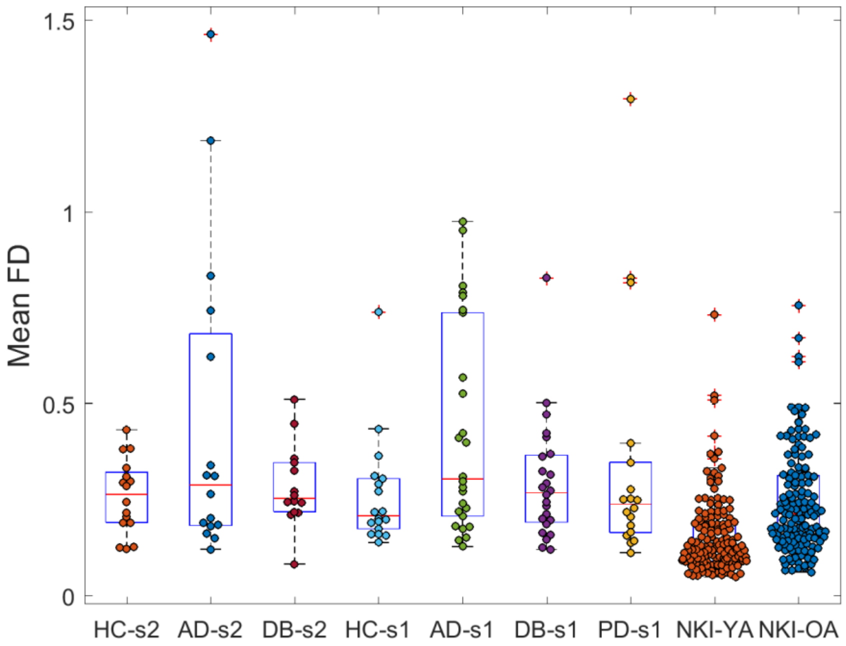

Supplementary Fig 9. Box-plots for mean FD (framewise displacement) values per group. Each participant's mean FD value is shown as small circles.

Subjects with a large head motion were excluded with a mean FD threshold of 0.5mm. The demographics of cohorts with FD exclusion are shown in Supplementary Table 2.

Supplementary Table 2. Study demographics with the exclusion of FD>0.5mm

	NKI		NCL			
	YA	OA	ADD	DLB	PDD	OA
N=290	N=148	N=142	N=28	N=35	N=14	N=33
Age	27.1(5.5)	68.5(4.9)	75.6(9.0)	77.7(6.7)	71.6(5.0)	76.2(6.9)
Sex	72M,76F	48M,94F	20M,8F	23M,12F	14M,0F	23M,10F
MMSE	--	--	21.5(3.9)	21.8(4.4)	24.2(2.9)	28.9(1.1)
CAMCOG	--	--	69.2(13.8)	73.2(13.3)	80.6(7.2)	96.5(3.4)
UPDRS III	--	--	3.1(3.4)	18.2(10.5)	25.6(7.7)	1.8(2.8)

All values are shown as mean (standard deviation)
N, number of participants; M, male; F, Female
MMSE, Mini-mental state examination
CAMCOG, Cambridge cognition examination
UPDRS III, Unified Parkinson's disease rating scale III
YA, young adults
OA, older adults
ADD, Alzheimer's disease dementia
DLB, dementia with Lewy bodies
PDD, Parkinson's disease dementia

To observe possible spurious correlations introduced by motion to the resting-state functional connectivity (RSFC), we generated QC-RSFC scatter plots, where QC stands for the motion statistic, mean FD in our case. FD-RSFC values were estimated by correlating mean FD and the inter-node Pearson's correlation across participants. Supplementary Fig 10 shows the FD-RSFC plots with the Euclidean distance between node pairs in the x-axis. For all cohorts, there was a mild correlation effect introduced by motion, where close-range connections were mostly affected. Motion effects on the long-range connections were on average zero. The right panel of Supplementary Fig 10 further displays the motion effect on mean FC strength in the y-axis. Edge distance was quantified and sorted by short (the top 20% close distance, smaller than 53mm), medium (20%~50%, about 53mm~80mm; 50%~80%, about 80mm~107mm) and long ranges (>80%, >107mm). Consistent with the previous work[3], the linear fit of this effect revealed that motion increased FC for short distances (NCL: Pearson, r=0.1850, p-

value=0.0337; NKI: Pearson, r=0.3194, p-value<0.001), but diminished connectivity or affected FC less for long distances (NCL: Pearson, r=0.0209, p-value=0.8123; NKI: Pearson, r=-0.1283, p-value=0.0270). The head motion was suggested to cause increased impact at short-range connections compared to longer ranges.

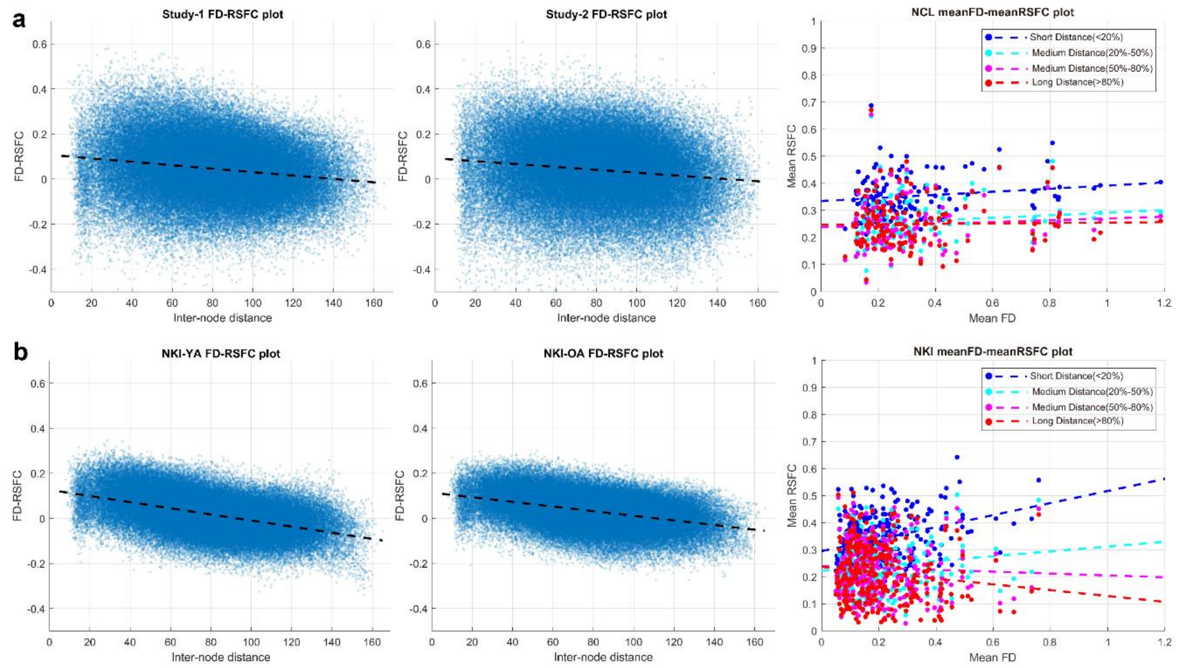

Supplementary Fig 10. Motion effect for the three study groups pre-processed in this study. **a** QC-RSFC plot with inter-node distance for Newcastle (NCL) cohort study 1 (left panel), QC-RSFC plot for NCL study 2 (medium panel) and correlation plot between mean FC and FD under different inter-node distance range for both two NCL groups (right panel). **b** QC-RSFC plot for NKI cohort of young adults (left panel), QC-RSFC plot for NKI cohort of older adults (medium panel) and correlation plot between mean FC and FD for both two NKI groups. The linear fits from left to right are: **a** Pearson, dark: r=-0.1728, p-value<0.001; dark: r=-0.1326, p-value<0.001; blue: r=0.1850, p-value=0.0337; cyan: r=0.1359, p-value=0.1204; magenta: r=0.0883, p-value=0.3141; red: r=0.0209, p-value=0.0337; **b** Pearson, dark: r=-0.4713, p-value<0.001; dark: r=-0.4046, p-value<0.001; blue: r=0.3194, p-value<0.001; cyan: r=0.1242, p-value=0.0324; magenta: r=-0.0433, p-value=0.4574; red: r=-0.1283, p-value=0.0270;

Due to the shown evidence of motion impact on short-range connections in YA, OA and neurodegenerative dementia groups, we repeated this analysis with mean FD added as a covariate of no interest together with sex and gender. In order to test changes after regressing out FD, we still used 451-ROI atlas for NKI and NCL cohorts at edge density 3.24%. As shown in Supplementary Fig 11a, the modularity statistic

of NCL group reaches a high value at edge density 3.24% compared with equivalent random networks, which is similar to the case without FD regression as shown in Supplementary Fig 2a. However, the modularity difference between ADD and other groups gets larger, which may be owing to the big ADD participant exclusion of high mean FD (shown in Supplementary Table 1, Table 2). Nevertheless, the eight consensus modules for NKI cohort remained (ref. Supplementary Fig 11b vs Fig 3a in the main text), while for NCL a total of eight modules (ref. Supplementary Fig 11c vs Fig 3c in the main text) were found in which several modules were consistent, such as cerebellum, motor-sensory, temporal and occipital modules. The MV pattern keeps the same with higher values at inferior frontal, parietal, insular cortices, inferior post-/pre-central gyri, superior temporal gyri, and with lower values at occipital, superior motor-sensory, temporal poles, cerebellum. The association cortices were found to have larger MV than primary cortices. As for MD (ref. Supplementary Fig 12 vs Supplementary Fig 3), identical to the analysis without FD regression, both NKI and NCL cohorts showed the pattern with low values in superior frontal, occipital, motor-sensory cortices while regions of high MD were located primarily in cerebellum, basal structures and insular cortices. The motion was demonstrated not to influence the main MV and MD distribution of low and high values shown in the foregoing analysis.

MV and MD comparison results between OA and YA groups for the NKI cohort are also consistent after regressing out FD as shown in Supplementary Fig 13 (versus Fig 4, 5 in the main text). The OA group showed higher MV within occipital, insular and ventral frontal cortices compared with YA, and lower MV within parietal, motor-sensory, cerebellum, part of the superior frontal cortices. The MD mostly decreased across the brain in OA except for the insulo-opercular cortex which showed significantly higher MD. The patterns from healthy ageing to dementia were also similar to the results before FD correction (ref. Supplementary Fig 14 vs Fig 6, 7 in the main text). Compared with OA, we found the PDD group had low differences for MV in most regions but significantly high differences for MD, especially in cerebellum, insulo-opercular and motor-sensory cortices. The other ADD and DLB groups showed differentiated patterns: high MV(MD) in occipital cortex (occipital and motor-sensory cortices) for ADD, while high MV/MD in occipital and motor-sensory cortices for DLB. From the global level, the mean MD across all nodes in dementia groups didn't change from OA either (Supplementary Fig 15b). The YA also had significantly higher mean

MD than OA, mainly in four of the eight modules: superior frontal-occipital, ventral frontal, temporal and cerebellum (Supplementary Fig 15c), which were mostly overlapped by results before mean FD regression. Locally, the MD patterns varied, however, were invariant to some degree. For instance, the MD change in the frontal related modules was on average negative in the dementia groups (Supplementary Fig 16e, h). And for the motor-sensory module, all groups remained a positive change relative to OA (Supplementary Fig 16a). Therefore, the additional motion processing was proved not to change the main patterns of how MV and MD change from healthy ageing to a dementia state due to neurodegeneration.

Overall, the above replication analysis indicated that our primary findings seemed robust to head motion although it did influence FC.

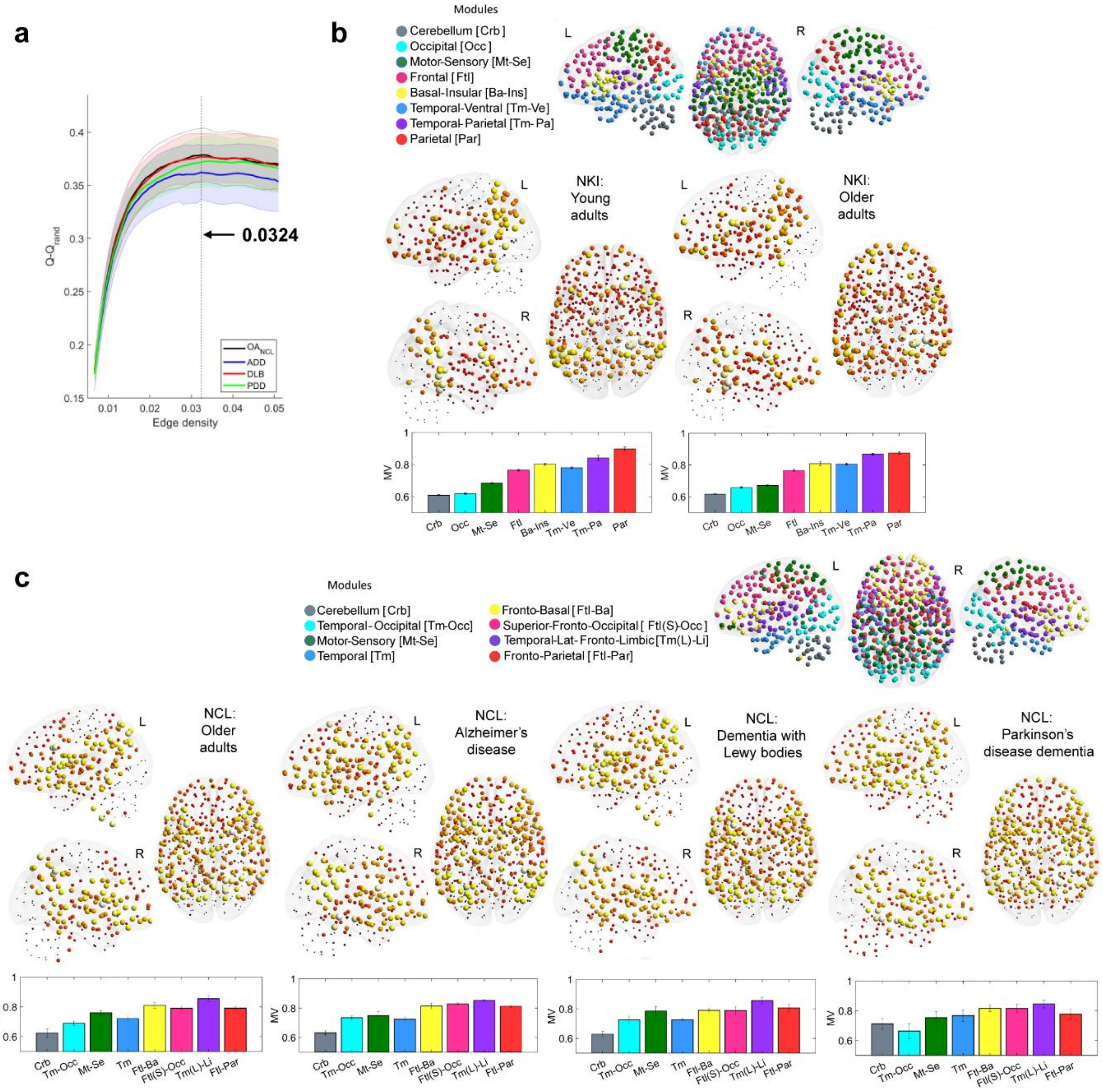

Supplementary Fig 11. Modular variability (MV), consensus communities and group means after regressing out FD. **a** Results presented here used the 451-ROI atlas for the NKI and NCL cohorts at edge density 3.24%. **b** Nathan Kline Institute (NKI) consensus modularity and module definitions shown in coloured spheres (top). Group mean MV for young adult (YA) and older adult (OA) groups (middle). Mean MV per module shown as bar plots for the OA and YA groups (bottom); colours for each bar match the communities. **c** Same as (**b**) for the Newcastle (NCL) cohort. Left hemisphere (L), right hemisphere (R). The mean values and standard deviations shown in the bar plots are obtained by 500 times with bootstrapping approach.

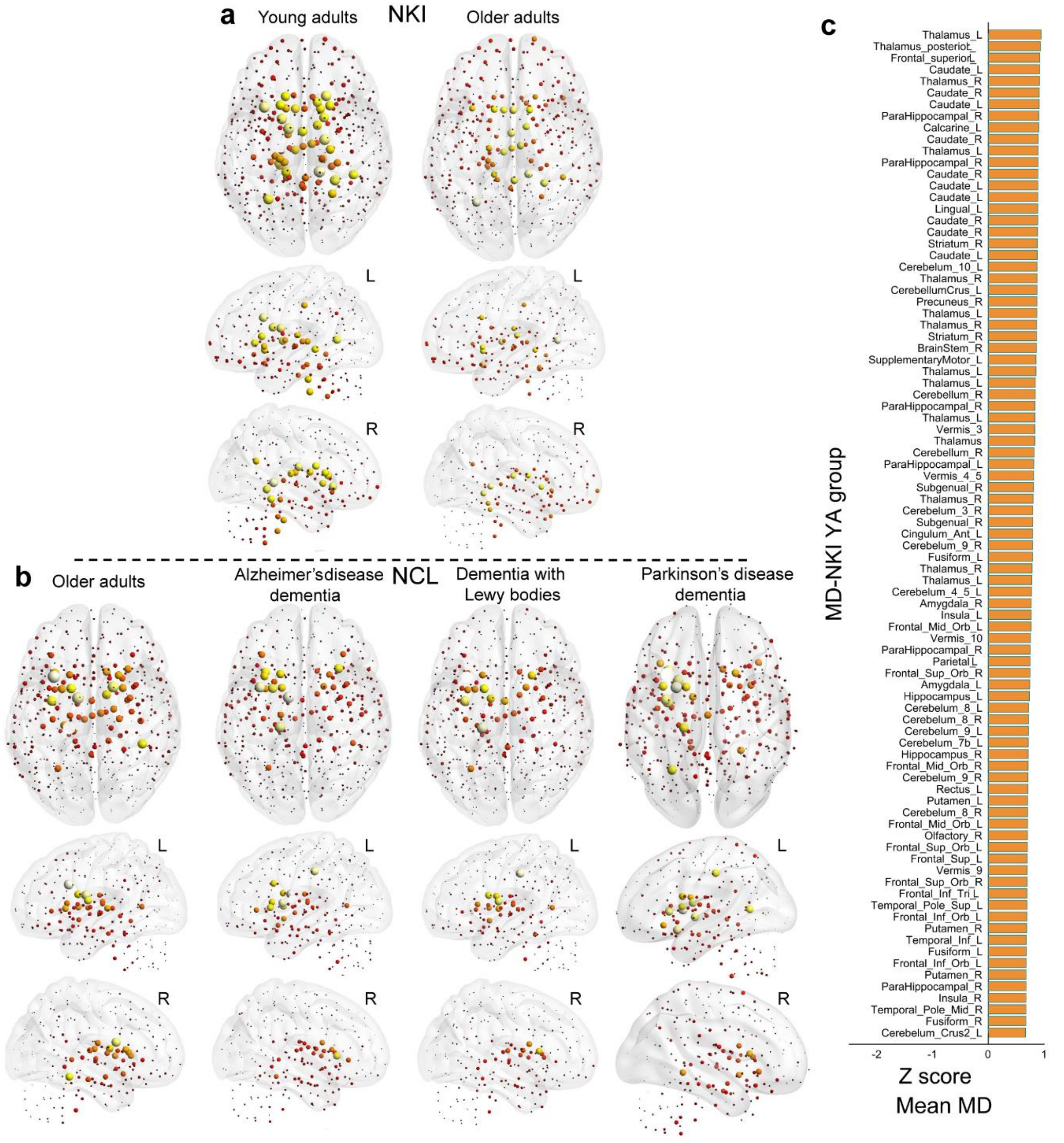

Supplementary Fig 12. Mean modular dissociation (MD) after regressing out FD. **a** Nathan Kline Institute (NKI) cohort MD values for the young adult (YA) and older adult (OA) groups. **b** Same as (**a**) for the Newcastle (NCL) cohort. **c** Strongest 20% of MD values within the NKI-YA group. Left hemisphere (L), right hemisphere (R). The results presented are from the 451-ROI atlas at optimal edge density.

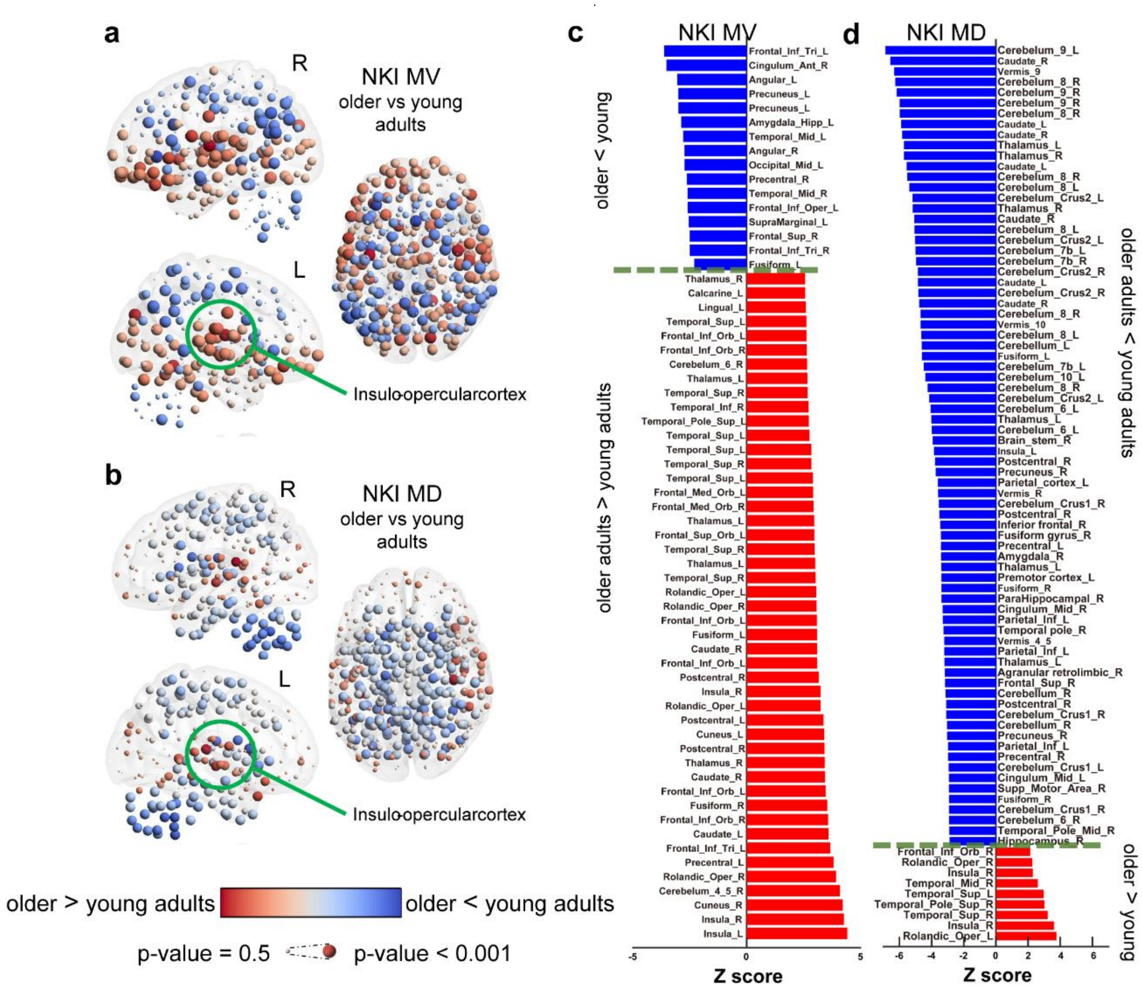

Supplementary Fig 13. Age effects of modular variability (MV) and modular dissociation (MD) after regressing out FD. **a** Age differences in MV within the Nathan Kline Institute (NKI) cohort, older adults (OA) compared with young adults (YA). **b** Age differences in MD within the NKI database, OA vs YA. **c** Significant brain regions from the NKI-MV comparison; corrected for multiple comparisons at p-value<0.05. **d** Significant brain regions for the NKI-MD comparison; corrected at p-value<0.05. Results were assessed with non-parametric permutations (5000) after regressing out covariates of no interest. Regions' names are given using the Automatic Anatomical Labelling (AAL).

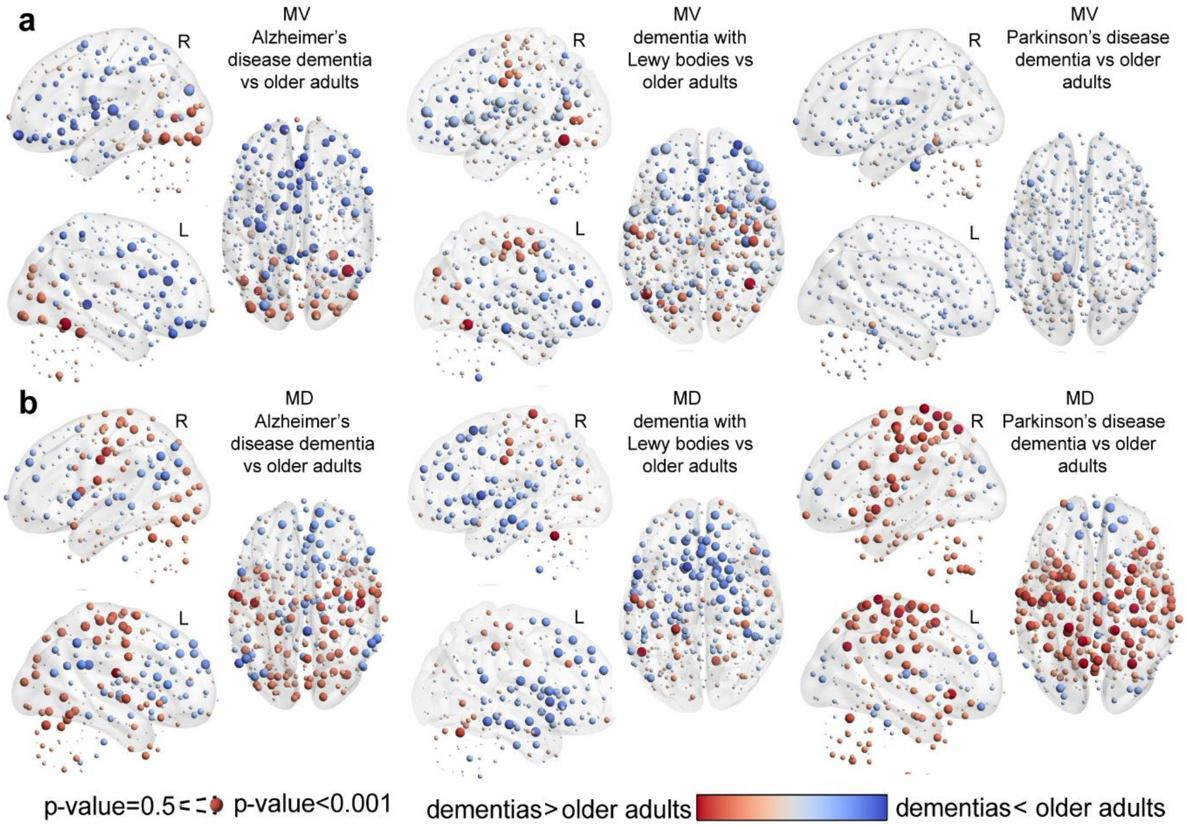

Supplementary Fig 14. Neurodegenerative dementia effects of modular variability (MV) and modular dissociation (MD) after regressing out FD. **a** MV differences between older adults (OA) and dementia groups: Alzheimer's disease dementia (ADD), dementia with Lewy bodies (DLB), and Parkinson's disease dementia (PDD). **b** MD differences between OA and dementias. Results were assessed with non-parametric permutations (5000) after regressing out covariates of no interest. Results for MV-PDD were not significant (uncorrected) while the rest of the comparisons showed significant uncorrected results. Only results for MD-PDD survived correction for multiple comparisons at a p-value < 0.05.

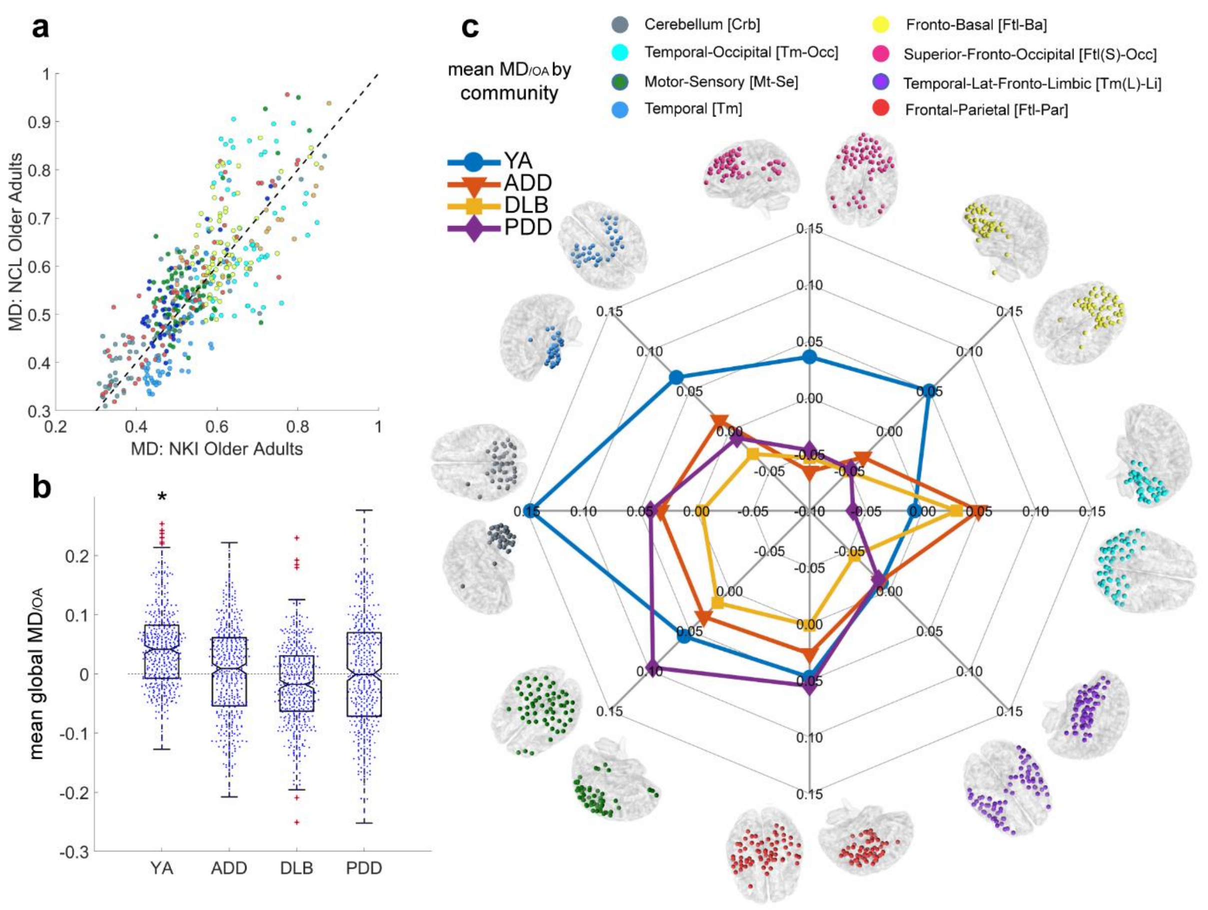

Supplementary Fig 15. Modular dissociation relative to older adults, $MD_{/OA}$; changes from healthy ageing to dementia after regressing out FD. **a** Scatter plot with MD values from the OA groups within the Nathan Kline Institute (NKI) and the Newcastle University (NCL) cohorts; colours are shown according to communities. Both OA groups were correlated; Pearson r=0.8, p-value = 6.68e-102, R^2=0.64. **b** Global $MD_{/OA}$ for the NKI-young adult (YA) group and the NCL groups; Alzheimer's disease dementia (ADD), dementia with Lewy bodies (DLB), and Parkinson's disease dementia (PDD). *Statistically different from zero, Wilcoxon signed rank test: p-value = 4.4329e-32. **c** Spider plot for Mean $MD_{/OA}$ by community. Ageing drives a significant decrease of MD shown by the high scores in YA group (blue line) and mainly for the cerebellum, temporal and superior frontal-occipital communities, suggesting that the ageing brain moves towards a local connectivity regime of segregation. Neurodegenerative dementia did not change overall brain's $MD_{/OA,}$ but alterations are present at different communities. See Supplementary Fig 16 for non-referenced results of MD values and box plots.

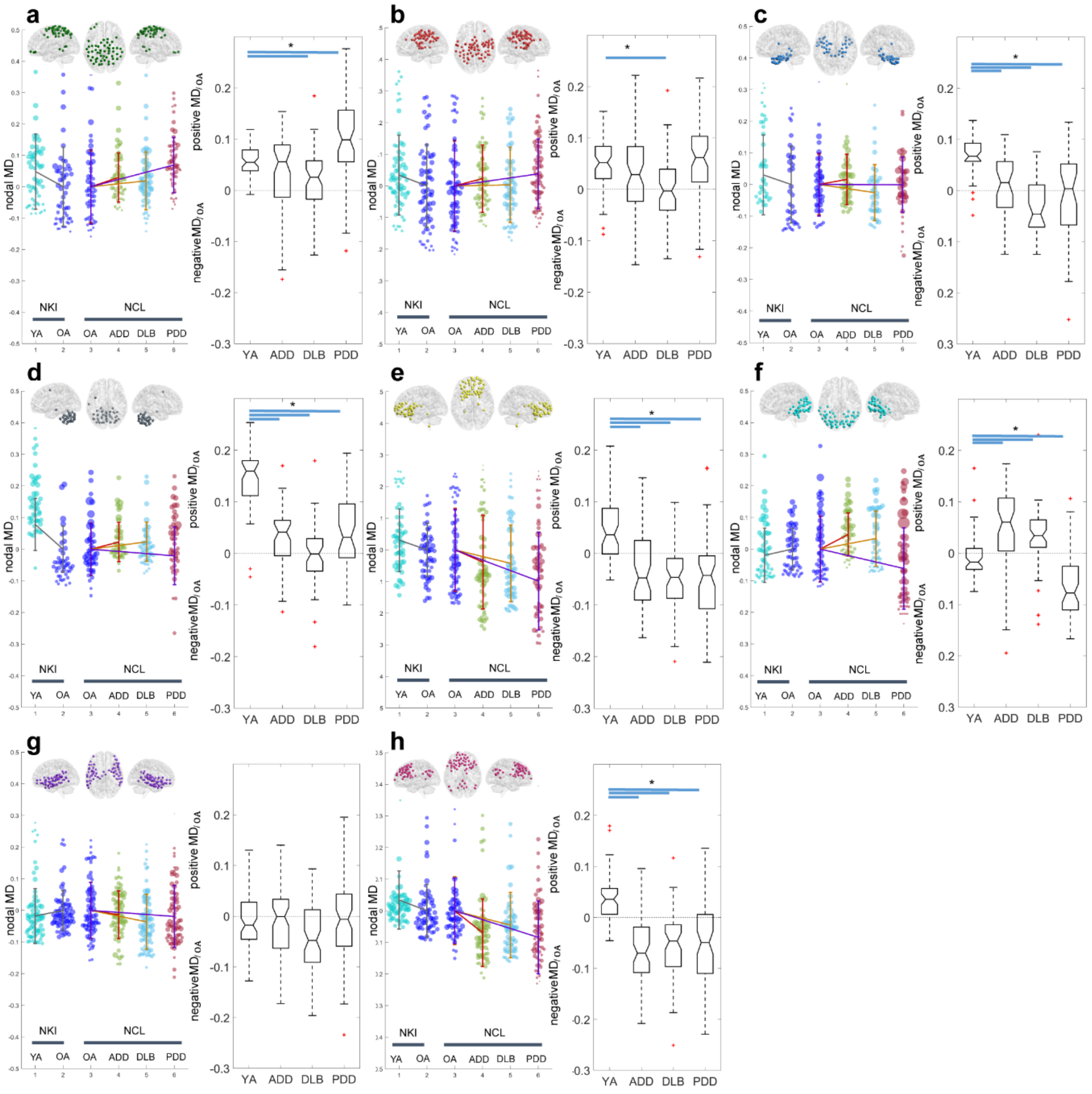

Supplementary Fig 16. Modular Dissociation (MD) values from the Nathan Kline Institute (NKI) and Newcastle University (NCL) databases after regressing out FD. MD values are shown as dotted violin plots. **a** Motor-Sensory, **b** Fronto-Parietal, **c** Temporal, **d** Cerebellum, **e** Frontal-Basal, **f** Temporal-Occipital, **g** Temporal-Lat-Fronto-Limbic, **h** Superior-Fronto-Occipital. Box plots with $MD_{/OA}$ values are also shown for each module. The mean values and standard deviations are derived from the whole module across nodes within the module.

Standard parcellation analysis

In the current study, we estimated and used four different functional parcellations derived from an independent study that was scanned in the same MRI. The use of in-house parcellations assure there is no bias introduced by including the same cohorts under analysis (NCL and NKI) for atlas estimation and no bias introduced by study site. Although evidence has shown that the measures of network segregation and integration seem to be robust to the underlying parcellations[4], it's unclear if our findings are also obvious in well-established brain parcellations. To this end, we conducted a replication analysis using a multi-modal atlas--Human Brainnectome Atlas[5] which in total included 210 cortical, 36 subcortical and 28 cerebellar subregions. The mean FD, sex, and age were added as covariates of no interest in analysis.

As shown in Supplementary Fig 17a, the optimal edge density was selected at 4.40% where there was a big bias as well between ADD and other groups because of high motion correction. The modular definitions were different from in-house parcellation studies (ref. Supplementary Fig 17b, c). For example, the frontal-parietal module in 451-ROI case was divided into two separate modules on the basis of Human Brainnectome Atlas for NKI cohort, and two separate modules in 451-ROI case: cerebellum and occipital modules were combined into one integrated module based on Human Brainnectome Atlas for NCL cohort. Nevertheless, the MV patterns were consistent with 451-ROI parcellation findings in which high MV in association cortices and low MV in primary cortices. More specifically, higher MV was found at inferior/superior frontal, parietal, basal structures, superior temporal gyri, and lower MV at occipital, superior motor-sensory, temporal poles, cerebellum. The MD also showed similar patterns (ref. Supplementary Fig 18): low values in superior frontal, occipital, motor-sensory cortices and high values located primarily in cerebellum, basal structures. The temporal cortex, in contrast, showed significantly high MD, especially for NKI cohort. Although this phenomenon was not obvious in 451-ROI NKI results, it was also found in the 177-ROI TFC (ref. Supplementary Fig 3) and 200-ROI NKI cases (ref. Supplementary Fig 6 b1-b4). Thus, despite differences derived from the standard atlas, the general MV and MD distributions of low and high values were maintained.

MV and MD comparisons between OA and YA groups were performed in NKI cohort, Supplementary Fig 19. Contrary to the 451-ROI studies, the patterns of high MV for

OA group shrunk, only the regions in insular, inferior frontal cortices were reserved. Several nodes of occipital cortex had a trend of high MV in OA as well but not significantly after FDR corrections while it was obvious in 451-ROI results. However, the patterns of low MV in OA which were primarily located in parietal, motor-sensory, cerebellum and part of the superior frontal cortices were consistent. Moreover, the results of MD comparison between OA and YA groups on the standard atlas were similar to 451-ROI NKI and 177-ROI TFC results, showing high MD in insulo-opercular cortex, part of frontal and occipital cortices although they were not significant after FDR correction. In general, the overall MV across the brain, on the contrary, decreased in OA. The overall MD, however, kept a decrease in OA. The key feature was coincident that the insulo-opercular cortex showed higher values of both MV and MD. Moving eyesight to the comparison between neurodegenerative dementias and OA group (Supplementary Fig 20), we came to the same conclusion that the PDD group had globally low differences for MV but significantly high differences for MD, especially in cerebellum, insulo-opercular and motor-sensory cortices. The ADD and DLB groups showed differentiated patterns even though the attached features were different from those in 451-ROI analysis. From the perspective of the global MD across all nodes, we found the mean MD higher in ADD group than in OA (Supplementary Fig 21b), which was not shown in 451-ROI case. But the mean MD in DLB and PDD groups didn’t change significantly from healthy ageing in the same way. Besides, the YA also showed higher global MD than in OA which was mainly in four of the seven modules: frontal-parietal, ventral frontal, temporal and temporal-occipital modules (Supplementary Fig 21c). Locally, the MD patterns varied but to some degree kept invariant compared to 451-ROI studies. For example, the MD change in the frontal related modules was also on average negative in the dementia groups (Supplementary Fig 22e, f). And for the motor-sensory module, all groups remained a positive change relative to OA (Supplementary Fig 22a). The standard-parcellation analysis consistently indicated a segregated modular structure with ageing and this pattern didn’t continue from healthy ageing to a dementia state, except for ADD which showed a further dissociation.

The differences between standard-parcellation and in-house parcellation analysis mainly existed in the MV comparison between YA and OA (Supplementary Fig 19a), and the mean MD comparison between ADD and OA (Supplementary Fig 21b). The

former differences could be caused by the bias of registration between standard parcellation and our study cohorts, which under different MRI scans and from different study sites. The other reason could be Human Brainnetome Atlas resolution including 274 ROIs compared to 451 ROIs of in-house atlas, which may weaken or decrease the significance because of the global mean across the region. The large similarity of decreased MV in OA group between results on Human Brainnectome Atlas and in-house 247-ROI atlas (Supplementary Fig 19a-b vs Supplementary Fig 19c-d) can support this. The later differences, on the one hand, could be owing to the confounding effect of head motion and the standard atlas. The global mean MD/OA for ADD group increased to be positive after regressing out head motion even though this was still not significant (Supplementary Fig 15b vs Fig 8b). The use of the Human Brainnetome Atlas promoted a further increase of the global mean MD/OA for ADD group which was even shown to be significantly greater than zero.

The above results demonstrated that the standard parcellation did affect the comparison results but the primary findings generally remained, especially for the large and small MV/MD distribution across the brain, the high MV/MD of OA group in the insulo-opercular cortex compared with YA, and local but not global changes of neurodegenerative dementia groups: DLB and PDD groups.

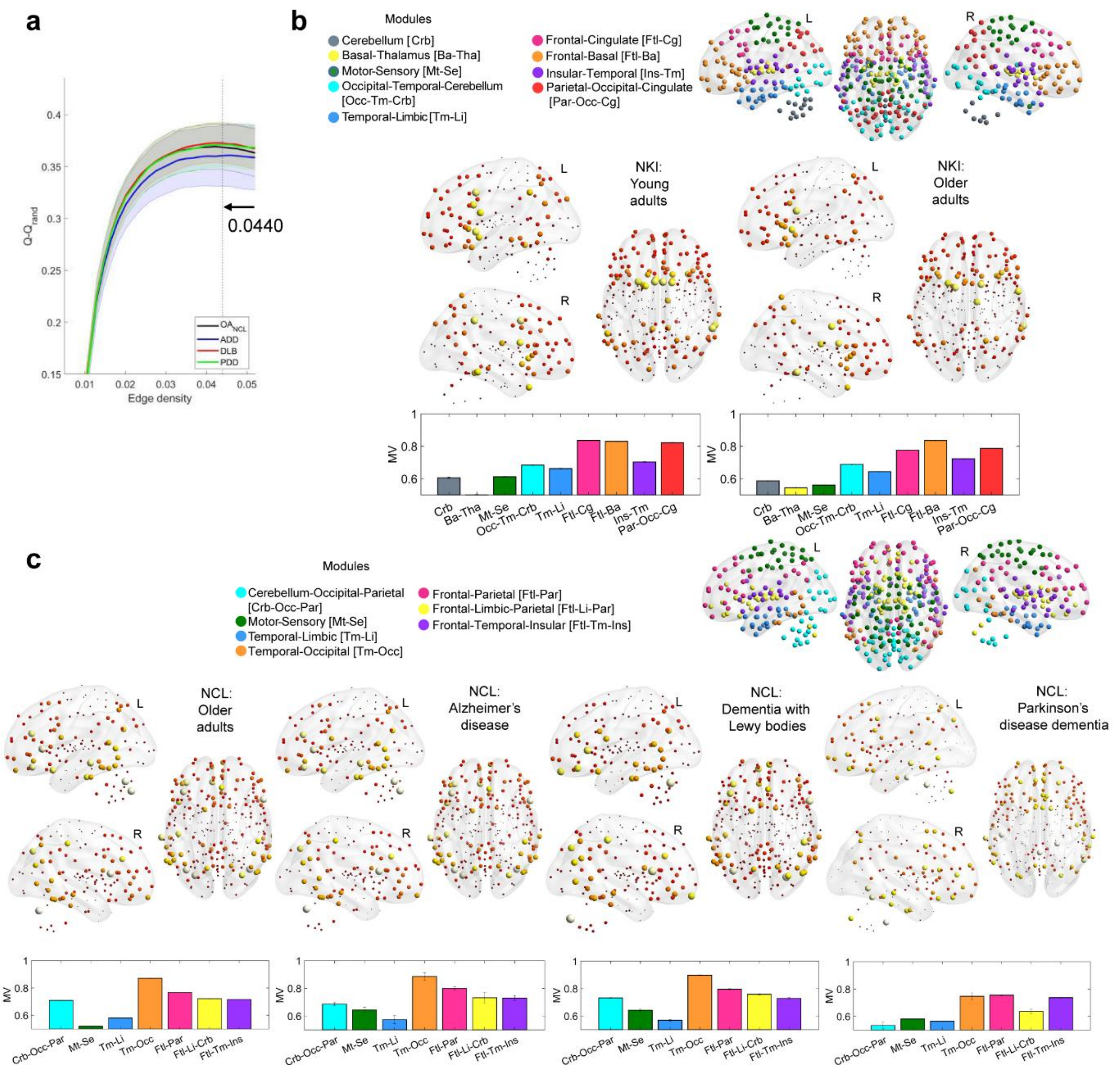

Supplementary Fig 17. Modular variability (MV), consensus communities and group means based on the Human Brainnetcome Atlas with 274 ROIs after regressing out FD. **a** Optimal edge density for the NCL cohort at 4.40%. **b** Nathan Kline Institute (NKI) consensus modularity and module definitions shown in coloured spheres (top). Group mean MV for young adult (YA) and older adult (OA) groups (middle). Mean MV per module shown as bar plots for the OA and YA groups (bottom); colours for each bar match the communities. **c** Same as (**b**) for the Newcastle (NCL) cohort. Left hemisphere (L), right hemisphere (R). Results presented here for the NKI and NCL cohorts are at optimal edge density. The mean values and standard deviations shown in the bar plots are obtained by 500 times with bootstrapping approach.

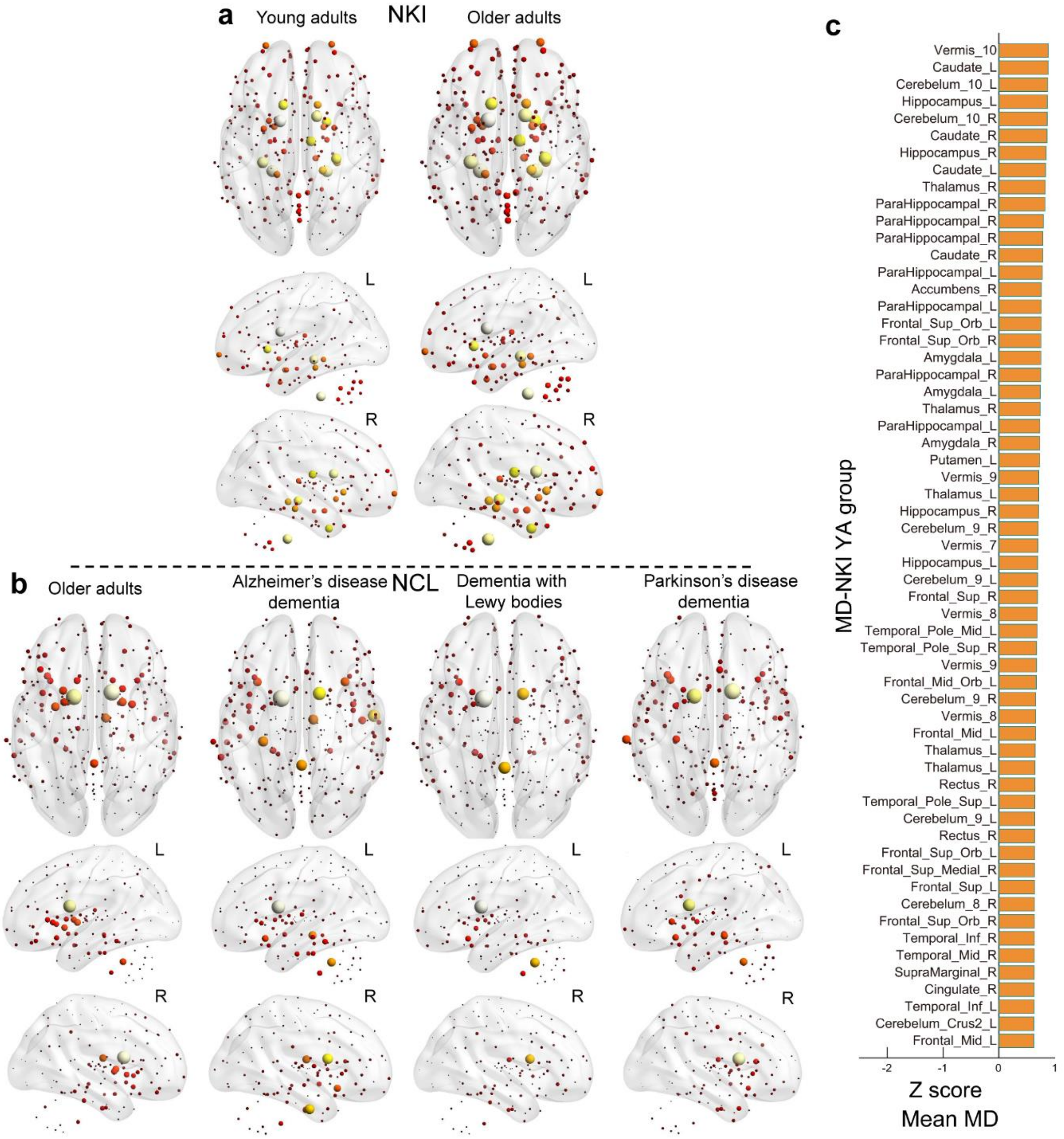

Supplementary Fig 18. Mean modular dissociation (MD) based on the Human Brainnetcome Atlas with 274 ROIs after regressing out FD. **a** Nathan Kline Institute (NKI) cohort MD values for the young adult (YA) and older adult (OA) groups. **b** Same as (**a**) for the Newcastle (NCL) cohort. **c** Strongest 20% of MD values within the NKI-YA group. Left hemisphere (L), right hemisphere (R). The results presented are at optimal edge density.

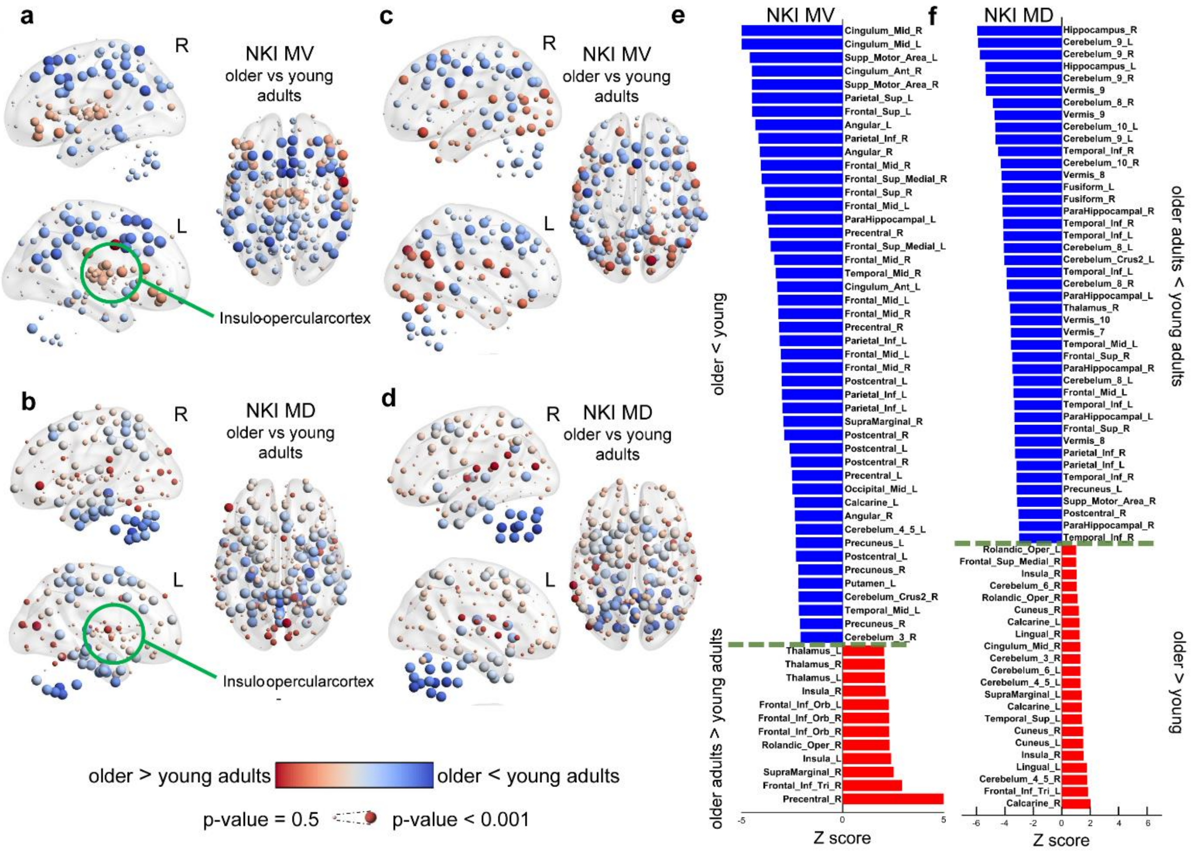

Supplementary Fig 19. Age effects of modular variability (MV) and modular dissociation (MD) based on the Human Brainnetcome Atlas with 274 ROIs after regressing out FD and based on the in-house 247-ROI atlas without FD regression. **a** Age differences in MV within the Nathan Kline Institute (NKI) cohort based on Human Brainnetcome Atlas, older adults (OA) compared with young adults (YA). **b** Age differences in MD within the NKI database based on Human Brainnetcome Atlas, OA vs YA. **c** Age differences in MV within the Nathan Kline Institute (NKI) cohort based on 247-ROI atlas, OA vs YA. **d** Age differences in MD within the NKI database based on 247-ROI atlas, OA vs YA. **e** Significant brain regions from the NKI-MV comparison based on the Human Brainnetcome Atlas; corrected for multiple comparisons at p-value<0.05. **f** Significant uncorrected brain regions for the NKI-MD comparison based on the Human Brainnetcome Atlas. Results were assessed with non-parametric permutations (5000) after regressing out covariates of no interest. Regions' names are given using the Automatic Anatomical Labelling (AAL).

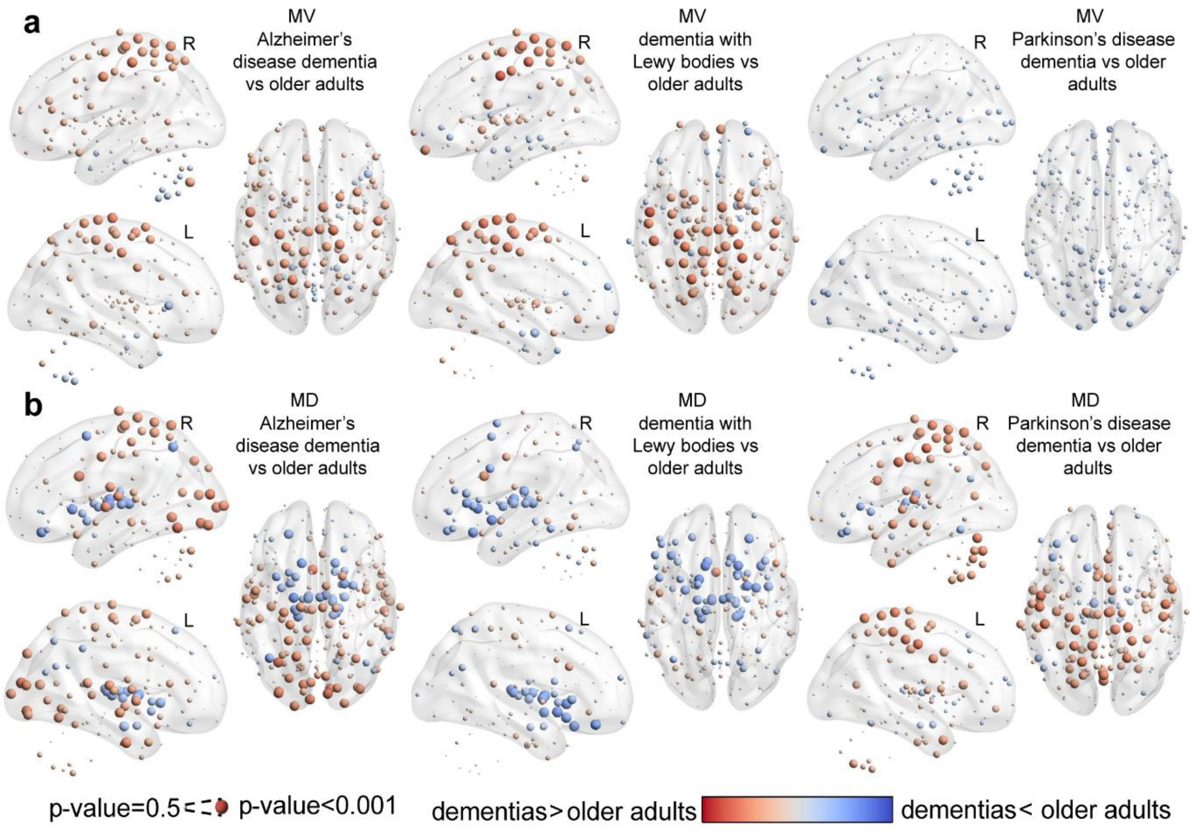

Supplementary Fig 20. Neurodegenerative dementia effects of modular variability (MV) and modular dissociation (MD) based on the Human Brainnetcome Atlas with 274 ROIs after regressing out FD. **a** MV differences between older adults (OA) and dementia groups: Alzheimer's disease dementia (ADD), dementia with Lewy bodies (DLB), and Parkinson's disease dementia (PDD). **b** MD differences between OA and dementias. Results were assessed with non-parametric permutations (5000) after regressing out covariates of no interest. Results for MV-PDD were not significant (uncorrected) while the rest of the comparisons showed significant uncorrected results. Results for MD-ADD, MD-PDD survived correction for multiple comparisons at a p-value < 0.05.

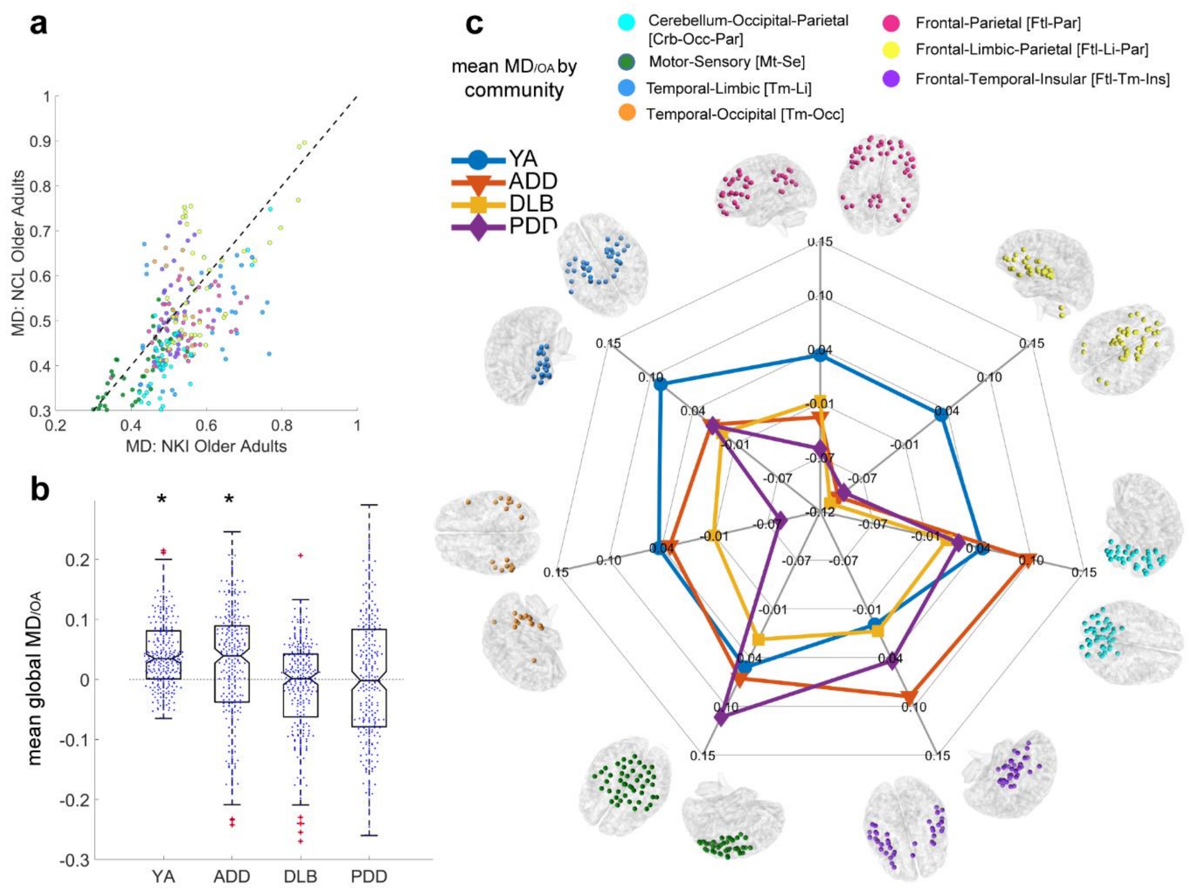

Supplementary Fig 21. Modular dissociation relative to older adults, $MD_{/OA}$; changes from healthy ageing to dementia based on the Human Brainnetcome Atlas with 274 ROIs after regressing out FD. **a** Scatter plot with MD values from the OA groups within the Nathan Kline Institute (NKI) and the Newcastle University (NCL) cohorts; colours are shown according to communities. Both OA groups were correlated; Pearson r=0.7, p-value = 4.26e-40. **b** Global $MD_{/OA}$ for the NKI-young adult (YA) group and the NCL groups; Alzheimer's disease dementia (ADD), dementia with Lewy bodies (DLB), and Parkinson's disease dementia (PDD). *Statistically different from zero, Wilcoxon signed rank test: p-value <0.001. **c** Spider plot for Mean $MD_{/OA}$ by community. Ageing drives a significant decrease of MD shown by the high scores in YA group (blue line) and mainly for the temporal and frontal-parietal communities, suggesting that the ageing brain moves towards a local connectivity regime of segregation. Neurodegenerative dementia, especially for DLB and PDD, did not change overall brain's $MD_{/OA,}$ but alterations are present at different communities. See Supplementary Fig 22 for non-referenced results of MD values and box plots.

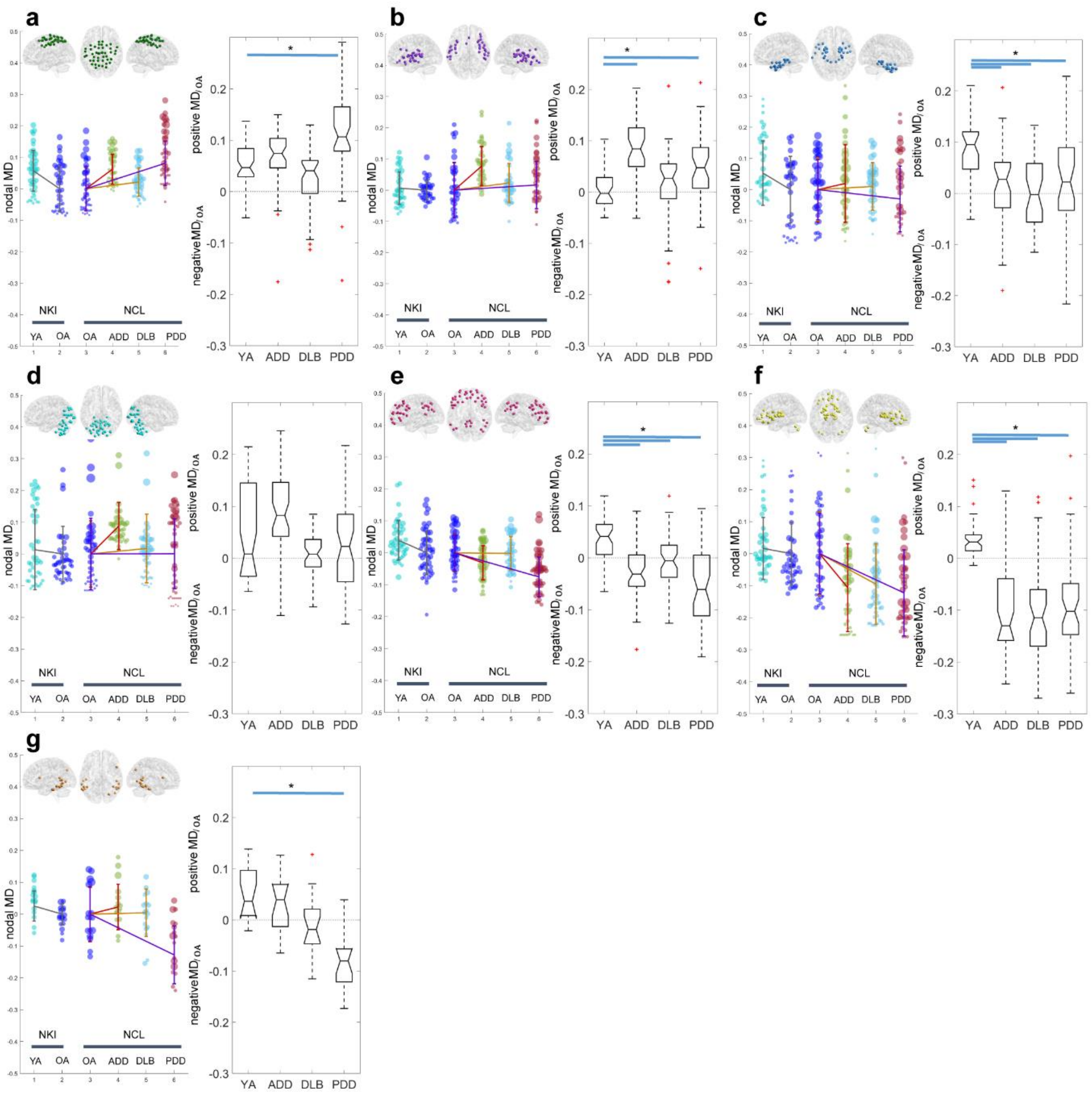

Supplementary Fig 22. Modular Dissociation (MD) values from the Nathan Kline Institute (NKI) and Newcastle University (NCL) databases based on the Human Brainnetcome Atlas with 274 ROIs after regressing out FD. MD values are shown as dotted violin plots. **a** Motor-Sensory, **b** Fronto-Temporal-Insular, **c** Temporal-Limbic, **d** Cerebellum-Occipital-Parietal, **e** Frontal-Parietal, **f** Frontal-Limbic-Parietal, **g** Temporal-Occipital. Box plots with MD$_{/OA}$ values are also shown for each module.

Limitations and considerations

Our investigation has some limitations and considerations that deserve to be mentioned. Previous research has pointed out that disease populations present with lower weights in their connectivity matrices[6]. We believe that our investigation is not affected by this factor because we compared communities (their variance and dissociation) and not connectivity strength or measures that are directly influenced by strength. Community estimation is only influenced by the distribution of weights within the connectivity matrix but not their global strength. Another related concern is proportional thresholding which is implemented to binarise weighted connectivity matrices. Currently, there is no consensus related to the treatment of weights from connectivity matrices, and other research groups have proposed different approaches, all of them with justifications. In our investigation, we decided to keep the negative Pearson correlation values because of the current research evidence indicating that these correlations are of biological origin, and we included their strength in our analyses by using the absolute value of the functional connectivity matrices. In a previous investigation from our research group[7], we implemented this approach and proved that it reproduced perfectly previous findings that used different weight treatment approaches, e.g. Alzheimer's disease patients showed a lower small-worldness when compared with age-matched control participants[7]. The use of the absolute value for the Pearson correlation matrix is similar to the edge estimation by wavelet correlations[8], whose coefficients are always positive. Hence, we are confident that our approach is robust and reproducible.

The patterns of mean group MD and MV found in our three independent databases (eight analysed groups) are very similar despite their differences in community structure; nine modules discovered for the NCL database, eight for NKI and seven in the TFC database. This suggests that MV and MD statistics are robust to small differences in community structure and that these reflected the phenomena under investigation: modular variability and dissociation.

A consideration during pre-processing of resting-state fMRI is the regression of the global signal. There is no current consensus about the regression of this variable; previous work has proved that this increases sensitivity in group comparisons and that it can reduce the influence of movement in fMRI time series[9]. However, it is also known

that this regression heavily modifies the weight distribution in the connectivity matrix which does affect community estimation[9]. For our in-house pre-processed databases, NKI and NCL, we did not implement global signal regression (we only regressed average CSF signal from bilateral ventricles). However, the TFC matrices downloaded from the UMCD had this regression. This may have contributed to the differences between the NCL-NKI cohorts and the TFC cohort (Fig 3-Fig 5 in the main text). In support of this, a recent investigation by Turchi, et al. [10] reported that the global signal is highly driven by basal activity. The authors performed pharmacological inactivation of two subregions of the nucleus basalis of Meyner (NBM). By this inactivation, Turchi, et al. [10] found that regional "global" signal (ipsilateral to the inactivated region) was suppressed, however, the presence of the resting state networks, such as the default mode network, remained invariant, proving that global signal in resting-state fMRI is of neuronal origin. It is possible therefore that global signal regression modifies brain connectivity at the basal brain by regressing out its activity. By this experiment, the authors have presented compelling evidence that discourages regression of the global signal in future resting-state fMRI studies.

Another consideration is the multi-site nature of the TFC database. The downloaded matrices came from nine different sites, and these were exclusive for older and younger adults; five sites comprised YA and four sites OA only. Because of this, we were only able to correct for within-group studies but not when comparing OA vs YA. We believe that the global higher MV (Fig 4 in the main text) and Q (Supplementary Fig 2) observed in OA compared with YA may be partially driven by study-site differences. However, the pattern of MV, MD and their differences between groups were remarkably similar in both NKI and TFC cohorts.

One last consideration in our investigation is that in order to observe the effects of neurodegenerative dementia in the old age brain, we used two independent databases; NKI for ageing and NCL for dementia. We normalised modular MD effects by their respective OA groups and studied the relative MD changes from the perspective of OA. This strategy allowed us to see that in healthy ageing, modular dissociation is increased at insular and occipital regions but decreased everywhere else and that in dementia there are deviations of MD at the modular level but not globally. This question could be ideally investigated with a longitudinal neuroimaging database that follows patients from youth to old age and then to dementia (if acquired). Databases

like this are currently under development although these will take some decades to be completed. For instance, the UK biobank multimodal neuroimaging database will be able to shed more light on this matter [11].

Supplementary References

1 Power, J. D., Barnes, K. A., Snyder, A. Z., Schlaggar, B. L. & Petersen, S. E. Spurious but systematic correlations in functional connectivity MRI networks arise from subject motion. NeuroImage 59, 2142-2154, doi:10.1016/j.neuroimage.2011.10.018 (2012).

2 Biswal, B. B. et al. Toward discovery science of human brain function. Proceedings of the National Academy of Sciences 107, 4734-4739, doi:10.1073/pnas.0911855107 (2010).

3 Satterthwaite, T. D. et al. Impact of in-scanner head motion on multiple measures of functional connectivity: relevance for studies of neurodevelopment in youth. Neuroimage 60, 623-632, doi: 10.1016/j.neuroimage.2011.12.063 (2012).

4 Arslan, S. et al. Human brain mapping: A systematic comparison of parcellation methods for the human cerebral cortex. NeuroImage 170, 5-30, doi:10.1016/j.neuroimage.2017.04.014 (2018).

5 Fan, L. , et al. "The Human Brainnetome Atlas: A New Brain Atlas Based on Connectional Architecture. Cerebral Cortex 26, 3508-3526, doi: 10.1093/cercor/bhw157 (2016).

6 van den Heuvel, M. P. et al. Proportional thresholding in resting-state fMRI functional connectivity networks and consequences for patient-control connectome studies: Issues and recommendations. NeuroImage 152, 437-449, doi:10.1016/j.neuroimage.2017.02.005 (2017).

7 Peraza, L. R., Taylor, J. P. & Kaiser, M. Divergent brain functional network alterations in dementia with Lewy bodies and Alzheimer's disease. *Neurobiology of aging* **36**, 2458-2467, doi:10.1016/j.neurobiolaging.2015.05.015 (2015).

8 Supekar, K., Menon, V., Rubin, D., Musen, M. & Greicius, M. D. Network Analysis of Intrinsic Functional Brain Connectivity in Alzheimer's Disease. *PLoS computational biology* **4**, e1000100, doi:10.1371/journal.pcbi.1000100 (2008).

9 Chai, X. J., Castanon, A. N., Ongur, D. & Whitfield-Gabrieli, S. Anticorrelations in resting state networks without global signal regression. *NeuroImage* **59**, 1420-1428, doi:10.1016/j.neuroimage.2011.08.048 (2012).

10 Turchi, J. *et al.* The Basal Forebrain Regulates Global Resting-State fMRI Fluctuations. *Neuron* **97**, 940-952.e944, doi:10.1016/j.neuron.2018.01.032 (2018).

11 Miller, K. L. *et al.* Multimodal population brain imaging in the UK Biobank prospective epidemiological study. *Nat Neurosci* **19**, 1523-1536, doi:10.1038/nn.4393 (2016).
