# Supplementary material for: The functional brain favours segregated modular connectivity at old age unless affected by neurodegeneration
Source: Commun Biol. 2021 Aug 16;4:973. doi: 10.1038/s42003-021-02497-0 (PMC8367990; doi:10.1038/s42003-021-02497-0)
Supplement: Supplementary file 3 — Description of Additional Supplementary Files [file 42003_2021_2497_MOESM3_ESM.pdf]

Description of Additional Supplementary Files

File name: Supplementary Data

Description: The supplementary data which contains all source data of graphs and chart in the manuscript.
