# Supplementary material for: The functional brain favours segregated modular connectivity at old age unless affected by neurodegeneration
Source: Commun Biol. 2021 Aug 16;4:973. doi: 10.1038/s42003-021-02497-0 (PMC8367990; doi:10.1038/s42003-021-02497-0)
Supplement: Supplementary file 5 — Reporting Summary [file 42003_2021_2497_MOESM5_ESM.pdf]

natureresearch

Corresponding author(s): Xue Chen, Joe Necus

Last updated by author(s): Jun 21, 2021

Reporting Summary

Nature Research wishes to improve the reproducibility of the work that we publish. This form provides structure for consistency and transparency in reporting. For further information on Nature Research policies, see Authors & Referees and the Editorial Policy Checklist.

Statistics

For all statistical analyses, confirm that the following items are present in the figure legend, table legend, main text, or Methods section.

n/a	Confirmed	
☐	☒	The exact sample size (*n*) for each experimental group/condition, given as a discrete number and unit of measurement
☐	☒	A statement on whether measurements were taken from distinct samples or whether the same sample was measured repeatedly
☐	☒	The statistical test(s) used AND whether they are one- or two-sided<br>*Only common tests should be described solely by name; describe more complex techniques in the Methods section.*
☐	☒	A description of all covariates tested
☐	☒	A description of any assumptions or corrections, such as tests of normality and adjustment for multiple comparisons
☐	☒	A full description of the statistical parameters including central tendency (e.g. means) or other basic estimates (e.g. regression coefficient) AND variation (e.g. standard deviation) or associated estimates of uncertainty (e.g. confidence intervals)
☐	☒	For null hypothesis testing, the test statistic (e.g. *F*, *t*, *r*) with confidence intervals, effect sizes, degrees of freedom and *P* value noted<br>*Give P values as exact values whenever suitable.*
☒	☐	For Bayesian analysis, information on the choice of priors and Markov chain Monte Carlo settings
☐	☒	For hierarchical and complex designs, identification of the appropriate level for tests and full reporting of outcomes
☐	☒	Estimates of effect sizes (e.g. Cohen's *d*, Pearson's *r*), indicating how they were calculated

Our web collection on statistics for biologists contains articles on many of the points above.

Software and code

Policy information about availability of computer code

Data collection	Matlab code will be available on-line through our lab's webpage, code examples will be placed in a GitHub account as well. Matrices will be available on our lab webpage; if space is not enough we will use the USC Multimodal Connectivity Database. For any of these options, links and information will be provided within our Lab's webpage www.lewybodylab.org.<br><br>Data sources are:<br>1) Enhanced Nathan Kline Institute (NKI) Rockland Sample, Nooner et al. (2012): http://fcon_1000.projects.nitrc.org/indi/enhanced/index.html<br>2) 1000 Functional Connectome (TFC) resting state matrices, Biswal et al. (2010): http://umcd.humanconnectomeproject.org<br>3) NKI functional connectivity matrices and atlases (brain parcellation), Newcastle University www.lewybodylab.org/research/modulardissociation.<br>4)The source data of graphs and charts are shown in Supplementary_Data.zip.
Data analysis	All software used within our study is stated in the Methods sections. With exception of Matlab, all software used is free and all Matlab toolboxes are open source. Software and algorithms used in our study were:<br><br>1) Modular dissociation and network construction by local threshold (Matlab code). Newcastle University www.lewybodylab.org/research/modulardissociation<br>2) Brain Connectivity Toolbox (BCT), Rubinov and Sporns (2010): https://sites.google.com/site/bctnet<br>3) Network Community Toolbox (NCT) – Consensus community, Doron et al. (2012): http://commdetect.weebly.com<br>4) BrainNet Viewer, Xia et al. (2013): https://www.nitrc.org/projects/bnv<br>5) FMRIB Software Library (FSL) v5.0 FMRIB, Oxford, UK: https://fsl.fmrib.ox.ac.uk<br>6) BrainWavelet Toolbox, Patel et al. (2014): http://www.brainwavelet.org<br>7) Anatomical location of regions in MNI coordinates; cuixufindstructure.m (Matlab) Xu Cui: http://www.alivelearn.net/<br>8) Diverging colour maps for visualization, Moreland (2009): http://www.kennethmoreland.com/<br>9) pyClusterROI for functional atlases from fMRI, Craddock et al. (2012): http://ccraddock.github.io/cluster_roi

10) Matlab 2017a Mathworks, Inc.: https://uk.mathworks.com
11) Python 2.7.12 Python: https://www.python.org

For manuscripts utilizing custom algorithms or software that are central to the research but not yet described in published literature, software must be made available to editors/reviewers. We strongly encourage code deposition in a community repository (e.g. GitHub). See the Nature Research guidelines for submitting code & software for further information.

Data

Policy information about availability of data

All manuscripts must include a data availability statement. This statement should provide the following information, where applicable:
- Accession codes, unique identifiers, or web links for publicly available datasets
- A list of figures that have associated raw data
- A description of any restrictions on data availability

Matlab code for MV, MD estimation and network construction by local thresholding are available at https://github.com/LuisPerazaRo/modulardissociation and by contacting the corresponding authors wmtmdlove@163.com, Joseph.necus@nottingham.ac.uk and senior authors Marcus.Kaiser@nottingham.ac.uk, john-paul.taylor@newcastle.ac.uk. The Enhanced Nathan Kline Institute (NKI) Rockland Sample is available at http://fcon_1000.projects.nitrc.org/indi/enhanced/index.html, and the 1000 Functional Connectome (TFC) resting state matrices are publicly available at http://umcd.humanconnectomeproject.org. The source data of Figure 2 to Figure 8 are shown in Supplementary_Data.zip.

Field-specific reporting

Please select the one below that is the best fit for your research. If you are not sure, read the appropriate sections before making your selection.

☒ Life sciences ☐ Behavioural & social sciences ☐ Ecological, evolutionary & environmental sciences

For a reference copy of the document with all sections, see nature.com/documents/nr-reporting-summary-flat.pdf

Life sciences study design

All studies must disclose on these points even when the disclosure is negative.

Sample size	The sample sizes resulted from all data available from the database sources, and after applying the age criteria for old and young adults. For the Newcastle databases we used all functional neuroimaging information available.
Data exclusions	We excluded participants whose structural MRI couldn't be coregistered correctly. These exclusions were stated in the Methods section: "After neuroimaging pre-processing, two AD and four DLB participants from the NCL and five OA participants from the NKI cohorts were excluded due to coregistration inaccuracies".<br><br>We also excluded participants who had a large head motion (mean framewise displacement (FD) >0.5mm) when we analyzed head motion effect on results. These exclusions were stated in the 'Motion analysis' section in Supplementary Material: "Subjects with large head motion were excluded with a mean FD threshold of 0.5mm. The demographics of cohorts with FD exclusion are shown in Table S2.".
Replication	(1) We tested two independent neuroimaging databases (NKI and 1000 functional connectome, TFC) and our results for the ageing effects were consistent in both cohorts. Importantly, the 1000-connectome database had an independent pre-processing pipeline than the NKI (which we processed locally) and despite this, results were consistent. (2) We also tested different atlases resolutions (100, 200 and 247 regions of interest) with different network edge densities or cost (optimal, 10% and 20%). For all these variables, mean MV and MD results remained invariant. (3) We finally tested head motion and standard parcellation (Human Brainnetome Atlas, 274 regions of interest) effects on two independent neuroimaging datasets (NKI and NCL) and our results showed that the primary results were robust although the modular definitions varied.
Randomization	Allocation of participants within each category was random, however covariates for sex, age and research site were used when possible.
Blinding	The ageing cohorts (TFC and NKI) were already collected but these were not divided in categories of age at the moment of collection; the original investigators aimed to have a broad sample of ages. For the neurodegenerative dementia cohorts (NCL), clinical diagnoses were needed and then collection is not blinded here. However, all data preprocessing was performed blinded to groups; e.g. young vs old.

Reporting for specific materials, systems and methods

We require information from authors about some types of materials, experimental systems and methods used in many studies. Here, indicate whether each material, system or method listed is relevant to your study. If you are not sure if a list item applies to your research, read the appropriate section before selecting a response.

Materials & experimental systems

n/a	Involved in the study
☒	☐ Antibodies
☒	☐ Eukaryotic cell lines
☒	☐ Palaeontology
☒	☐ Animals and other organisms
☐	☒ Human research participants
☒	☐ Clinical data

Methods

n/a	Involved in the study
☒	☐ ChIP-seq
☒	☐ Flow cytometry
☐	☒ MRI-based neuroimaging

Human research participants

Policy information about studies involving human research participants

Population characteristics

Only age and gender were used as populations covariates and these are applied as standard in functional MRI studies. We additionally included the study/site covariate when possible to account for study/site differences. Population characteristics are described in the demographic Table S1 in the Supplementary Information. Participants were grouped as follows:

NKI:
  Young adults: N = 151, age: 27.1 (5.4), sex: 74M, 77F.
  Older adults: N = 146, age: 68.7 (5.0), sex 48M, 98F.
TFC:
  Young adults: N = 257, age: 22.9 (4.35), sex: 102M, 145F.
  Older adults: N = 102, age: 58.7 (9.0), sex: 44M, 58F.
NCL:
  Alzheimer's (ADD): N = 42, age: 77.2 (8.6), sex: 30M, 12F.
  Lewy bodies (DLB): N= 38, age: 77.6 (6.4), sex: 25M, 13F.
  Parkinson's (PDD): N = 17, age: 71.8 (5.0), sex: 17M, 0F.
  Older adults (OA): N = 34, age: 76.4 (7.0), sex: 24M, 10F.

Recruitment

For the Newcastle databases, two independent neuroimaging databases were combined from two clinical studies. Participants in these studies were recruited within the north-east of England and patients with neurodegenerative dementia were contacted through old-age psychiatry and neurology services in Newcastle area (United Kingdom). A total of 42 dementia with Lewy bodies (DLB, N=16 in study 1 and N=24 in study 2), 44 Alzheimer's disease dementia (ADD, N=16 in study 1 and N=28 in study 2) and 17 Parkinson's disease dementia (PDD, N=17 in study 2 only) patients were recruited. Additionally, 34 age-matched healthy control participants (N=16 in study 1 and N=18 in study 2) were recruited as a comparison group. All patients were diagnosed by two experienced clinicians according to the clinical criteria for these diseases.

For the recruitment of the NCL participants, there was no bias other than the known diagnosis performed by the clinical expert.

The other two other ageing cohorts (NKI and the 1000 functional connectome) are standard cohorts that have been investigated in several previous studies, and their recruitment is described in open access publications: Nooner et al. (2012) and Biswal et al. (2010).

Ethics oversight

For both studies, approval was granted by the Newcastle Ethics Committee and all participants gave informed consent.

Note that full information on the approval of the study protocol must also be provided in the manuscript.

Magnetic resonance imaging

Experimental design

Design type

Resting state functional MRI

Design specifications

For the Newcastle cohorts, 128 images at a TR of 3000 ms were recorded after scanner stabilization.

Behavioral performance measures

Because this is a resting state study, no behavioral measures were investigated.

Acquisition

Imaging type(s)

functional MRI

Field strength

3 Tesla (Newcastle)

Sequence & imaging parameters

Acquisition protocol used a magnetisation prepared rapid gradient echo (MPRAGE) sequence, sagittal acquisition, echo time 4.6 ms, repetition time 8.3 ms, inversion time 1250 ms, flip angle 8°, SENSE factor = 2, and an in-plane field of view of 256×256 mm2 with a slice thickness of 1.2 mm yielding a voxel size of 0.93 × 0.93 × 1.2 mm^3 for study 1. For study 2, an in-plane field of view of 240 × 240 mm^2 with a slice thickness of 1.0 mm yielding a voxel size of 1.0×1.0×1.0 mm^3 was used. For functional resting-state neuroimaging, both studies used the same recording protocol: gradient

echo echo-planar imaging sequence with 25 contiguous axial slices, 128 volumes, anterior-posterior acquisition, in-plane resolution = 2.0 × 2.0 mm^2, slice thickness = 6 mm, repetition time = 3000 ms, echo time = 40 ms, and field of view = 260 × 260 mm^2.

Area of acquisition: Whole brain

Diffusion MRI: ☐ Used ☒ Not used

Preprocessing

Preprocessing software: Neuroimages from the NCL and NKI databases were pre-processed with the same pipeline. Non-brain tissue was stripped with the brain extraction tool (BET) from the FMRIB Software Library (FSL version 5.0) and results visually inspected to check if brain structures were complete and isolated. Resting-state functional MRI (rs-fMRI) were then processed. For the NKI resting state neuroimages, the first five volumes were deleted because the time series were not steady. Structural and functional images were then coregistered using FEAT (part of the FSL toolbox).

Normalization: Nonlinear coregistration to MNI152 space was implemented with the FMRIB's Nonlinear Image Registration Tool (FNIRT) within FSL. Then functional images were transformed to a 4×4×4 mm^3 resolution, and as final step, a 6-mm full-width half maxima (FWHM) spatial smoothing was applied to all volumes.

Normalization template: The FSL software used the MNI152 standard.

Noise and artifact removal: The most important artifact in fMRI is movement within the MRI. Our preprocessing protocol included three data processing steps dedicated to reduce the effects of movement, if any:

For the NCL and NKI cohorts, all rs-fMRIs were motion corrected using the FMRIB's Linear Image Registration Tool (FLIRT) without spatial smoothing. Then, the six-movement variables from FLIRT and the average time series from the bilateral ventricle were regressed out from all resting state images. In order to further correct for movement and other artifacts, the rs-fMRI time series were despiked with the BrainWavelet Toolbox; parameters were adjusted to despike approximately 0.6% of the times series. Finally, time series were high-pass filtered with a 150-second filter and smoothed.

To further test the movement effect on our results, we did an replication analysis in which we excluded participants whose mean FD were larger than 0.5mm and added the mean FD as a covariate of no interest. The comparison results demonstrated that after above movement preprocessing, the head motion did not influence our primary findings. The mean MV and MD results were consistent.

For the 1000-functional-connectome cohorts, the connectivity matrices were downloaded from the USC Multimodal connectivity database and hence these data were preprocessed elsewhere by the original authors; Biswal et al. (2010).

Volume censoring: Structural MRIs whose coregistration was problematic was excluded. This is mentioned in the Methods section:

"After neuroimaging pre-processing, two AD and four DLB participants from the NCL and five OA participants from the NKI cohort were excluded due to coregistration inaccuracies".

Statistical modeling & inference

Model type and settings: Structural MRIs whose coregistration was problematic was excluded. This is mentioned in the Methods section:

"After neuroimaging pre-processing, two AD and four DLB participants from the NCL and five OA participants from the NKI cohort were excluded due to coregistration inaccuracies".

Effect(s) tested: Only multivariate regressions and two-sample t-tests (two tailed) were implemented. p-values from these tests were assessed and reported.

Specify type of analysis: ☐ Whole brain ☐ ROI-based ☒ Both

Anatomical location(s): Locations for the network connectivity analysis were defined by functional atlases; 100, 200, 247 and 451 regions of interests. These atlases will be available as part of the data sharing statement for reproducibility. One additional standard atlas used in the standard parcellation replication analysis--Human Brainnetome Atlas includes 210 cortical, 36 subcortical and 28 cerebellar subregions in total which is freely available for download at http://atlas.brainnetome.org.

Statistic type for inference (See Eklund et al. 2016): In our investigation we performed network analysis from which we extracted two statistics: Modular Variability (MV) and Modular Dissociation (MD).

Correction: FDR (with the Benjamini and Hochberg procedure in Matlab; mafdr.m function at p-value < 0.05 for significance level) correction was applied to all regional results for MD and MV differences between two groups. When results did not survived this correction, this is reported in our manuscript.

Models & analysis

n/a	Involved in the study
☐	☒ Functional and/or effective connectivity
☐	☒ Graph analysis
☐	☒ Multivariate modeling or predictive analysis

Functional and/or effective connectivity

Connectivity measures for all three databases were Pearson's correlations; these were used as weights for the connectivity matrices.

Graph analysis

Brain networks were thresholded by edge density (using two network construction methods) but edge weights (the absolute value of the Pearson's correlations) were preserved for community estimation with Louvain's algorithm. After this, community definitions at the subject level were the base for all subsequent analyses with Modular Dissociation (MD) and Modular Variability (MV).

Multivariate modeling and predictive analysis

In the multivariate regressions for strength versus Euclidean distance, weight strength was the dependent variable with the following model:

strength ~ β1·distance + β2·group + β3·group*distance + β4·age + β5·sex + β6·study +1, where the coefficients β2 and β3 account for group differences in intercept and slope respectively.

Nodal MV differences between groups were assessed with nonparametric permutations (5000 permutations) after regressing out age, sex and study covariates. For the NKI and TFC cohorts, the age covariate was demeaned within groups only in order to correct for age variances without losing the group effect (YA vs OA). For the TFC cohort, study covariates could only be regressed within OA and within YA groups since neuroimaging acquisition for these two groups were recorded at different sites. For the NCL cohort we added the study covariate, and the NKI analysis did not use a covariate for different studies.

Differences between groups for MD were assessed directly with nonparametric permutations (5000 permutations) after regressing out age, sex and study covariates in a similar fashion as MV.

In the replications of head motion and standard parcellation effects on our results, the mean FD was added as one covariate of no interest. And the nonparametric permutations (5000 permutations) were also used in group comparisons after regressing out age, sex, study covariates and mean FD.
